# A New Synthesis of Enantiopure Amine Fragment: An Important Intermediate to the Anti-HIV Drug Lenacapavir

**DOI:** 10.1021/acs.joc.4c02380

**Published:** 2024-12-16

**Authors:** Anand H. Shinde, Ramakrishna Sayini, Piyal Singh, Justina M. Burns, Saeed Ahmad, G. Michael Laidlaw, B. Frank Gupton, Douglas A. Klumpp, Limei Jin

**Affiliations:** Medicines for All Institute, Virginia Commonwealth University, Richmond, Virginia 23284-3068, United States

## Abstract

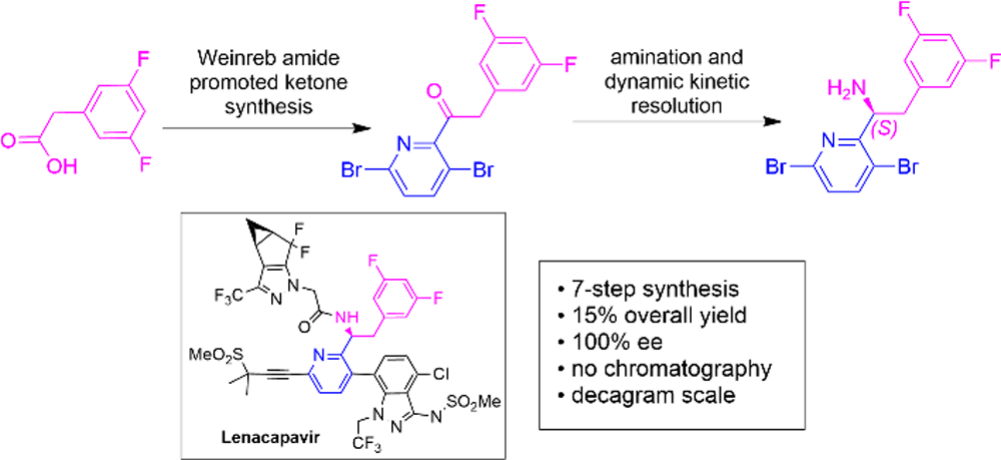

Herein, we describe a new seven-step approach to prepare (*S*)-1-(3,6-dibromopyridin-2-yl)-2-(3,5-difluorophenyl)ethan-1-amine ((*S*)-**4**) from the inexpensive 2-(3,5-difluorophenyl)acetic acid. The key steps in the sequence include (1) the Weinreb amide-based ketone synthesis to provide an entry point to the core structure; (2) simple functional group transformations to afford the racemic amine **4**-*rac*; and (3) dynamic kinetic resolution (DKR) to access the chiral amine (*S*)-**4**. This seven-step process delivered the enantiopure amine (*S*)-**4** in an overall isolated yield of approximately 15%. The process was demonstrated on a decagram scale, and the process requires no chromatographic purifications. Single-crystal X-ray crystallography measurements verified the chiral amine structure and absolute configuration.

## Introduction

The human immunodeficiency virus (HIV) remains one of the most serious health threats in the world. With its progression to acquired immunodeficiency syndrome (AIDS), it is estimated that there are annually over 600,000 deaths worldwide from this disease and over 40 million deaths since the start of the epidemic.^1^ There are currently about 40 million people globally who are infected by HIV, including 1.5 million children, and there are more than 1 million new infections annually.^2^ Among the most promising therapies for the treatment of HIV infections, lenacapavir is a first-in-class drug that targets the HIV capsid protein.^3−5^ It disrupts the functioning of the capsid protein across multiple steps in the viral life cycle. Remarkably, this substance has shown activity against all subtypes of HIV-1, including multidrug-resistant strains. This activity has been demonstrated at picomolar concentrations.^6^ With its long-acting properties and bioavailability for oral and injectable dosing, lenacapavir is likely to become a first-line treatment for HIV infections.

**Figure 1:**
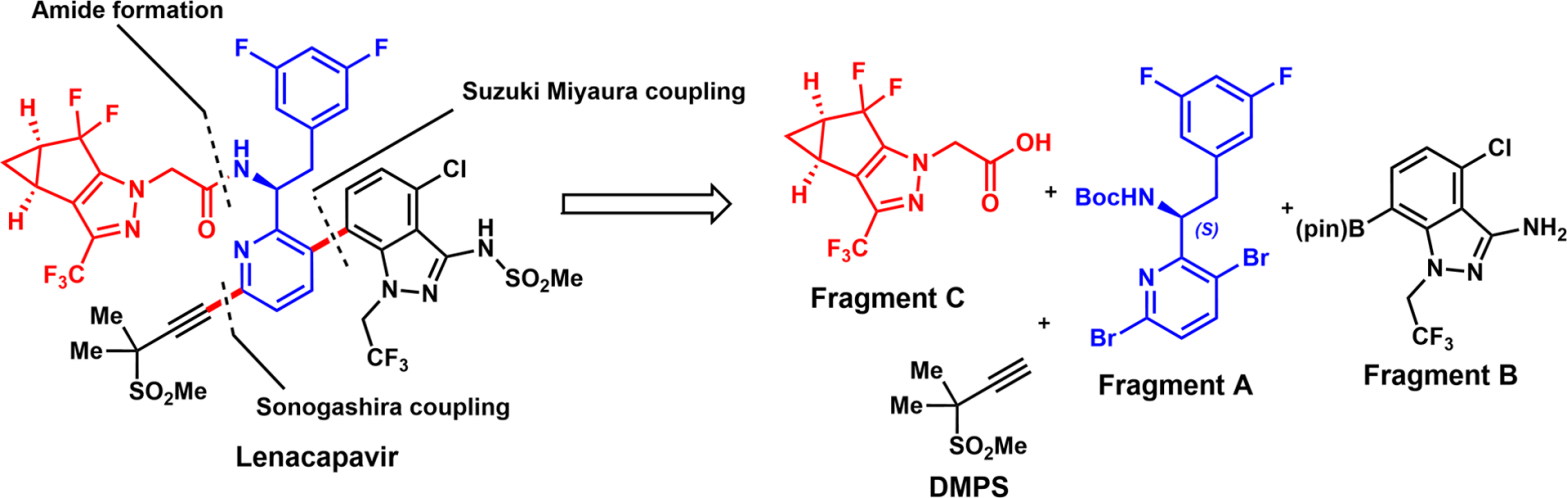
Retrosynthetic disconnections for lenacapavir and its simpler constituents for chemical synthesis.

Lenacapavir was first reported by Gilead Sciences in a family of patents and publications in 2018–2020.^7−10^ The Gilead synthesis of lenacapavir involves the convergence of three major fragments and a propargyl sulfone (DMPS, Figure 1). The central fragment of lenacapavir is Fragment A (a Boc-protected chiral amine), which is joined to Fragment B using a Suzuki–Miyaura coupling and to Fragment C using a HATU-promoted amide coupling. Fragment A is coupled to DMPS using a Pd-catalyzed Sonogashira reaction.

**Scheme 1:**
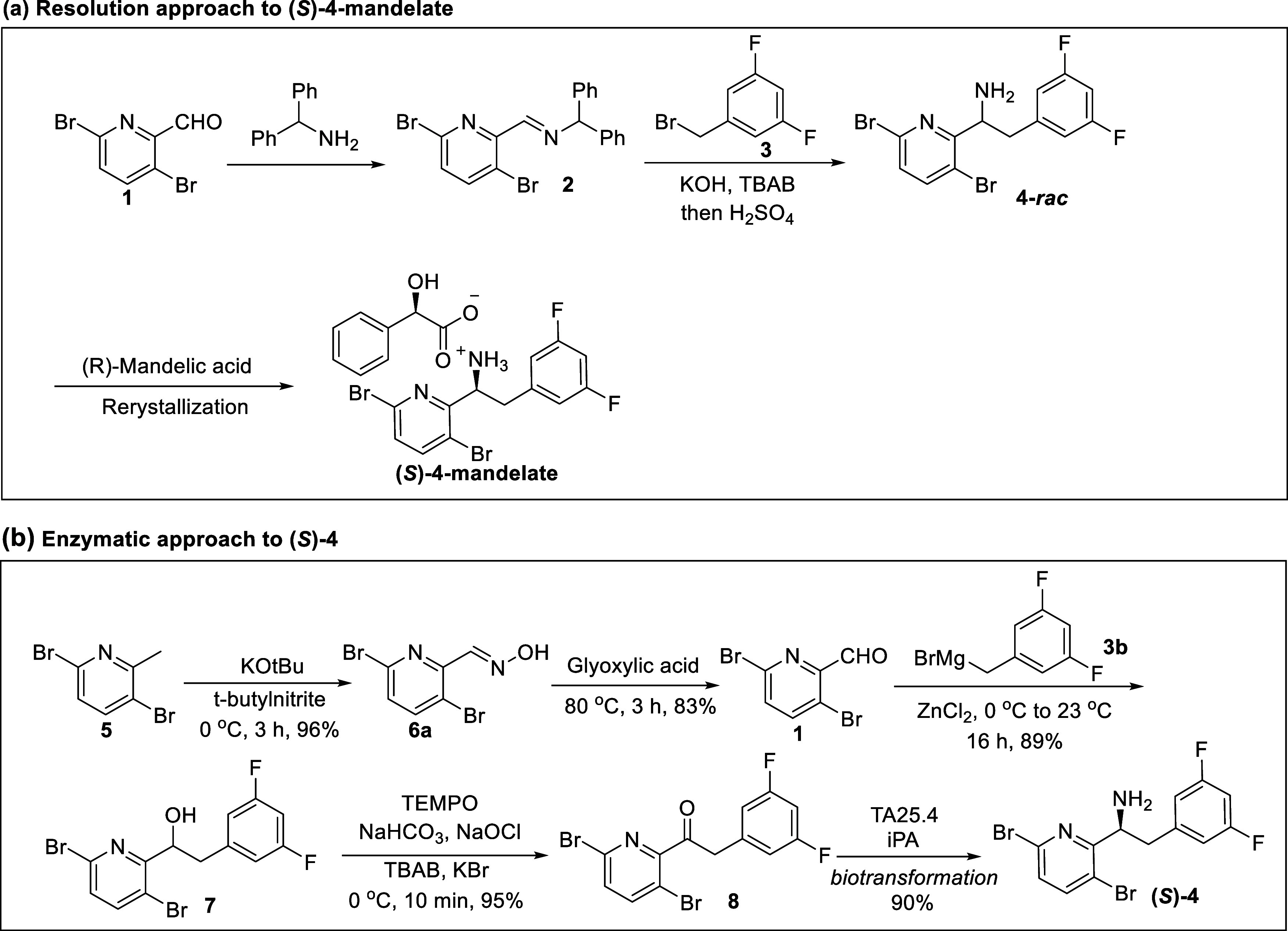
Methods for the Synthesis of Fragment A and Related Conversions (a) Resolution approach to (*S*)-4-mandelate (Reproduced from ref (11). Available under a Creative Commons Attribution 4.0 International License); (b) Enzymatic approach to (*S*)-4 (Reproduced from ref (12) Copyright [2024] American Chemical Society).

**Scheme 2:**
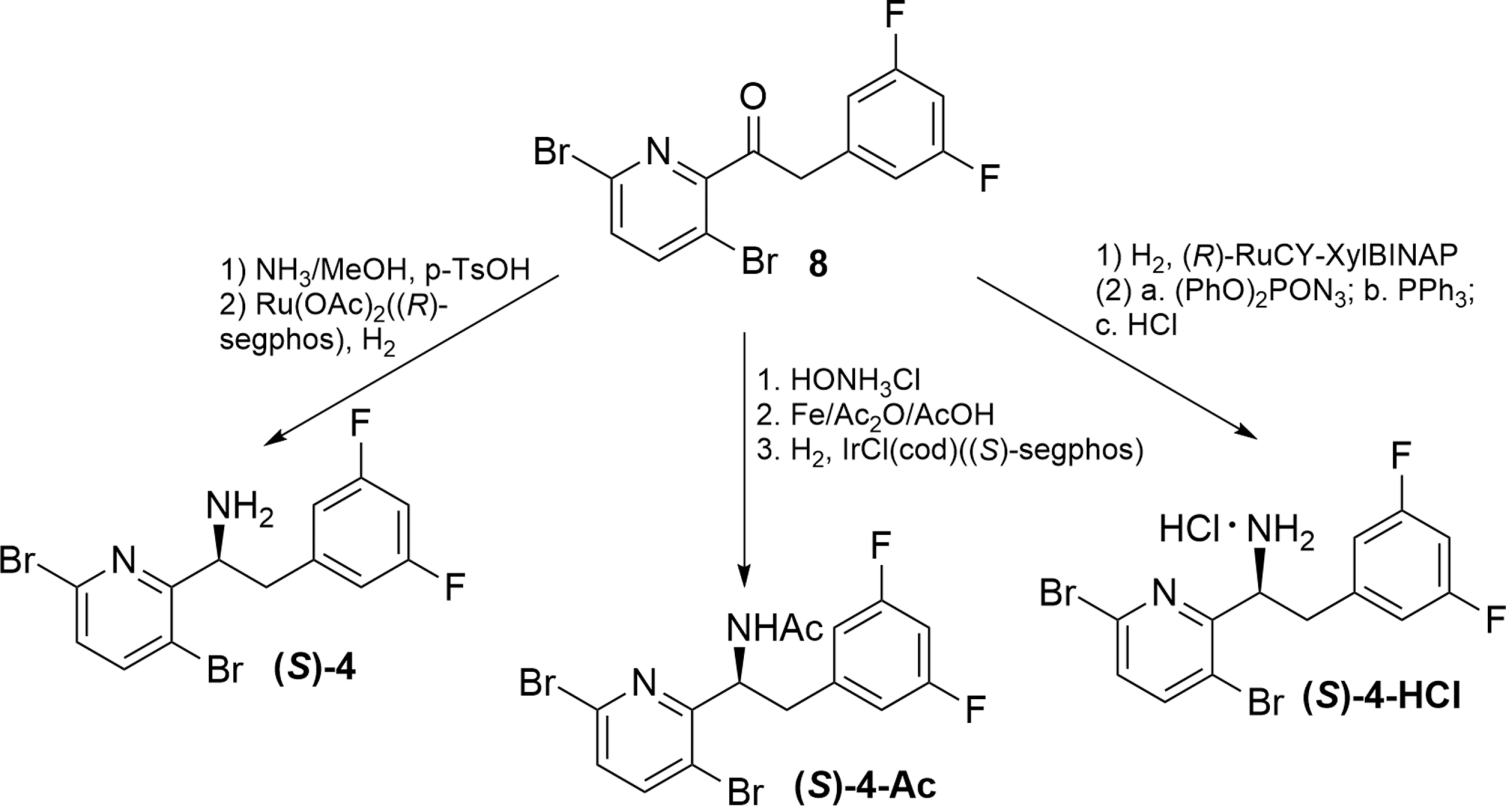
Reported Transformations of Ketone **8** to Chiral Amine and Derivatives

Synthesis of racemic amine **4**-*rac* was achieved by the alkylation of imine **2** with 3,5-difluorobenzyl bromide **3**. Resolution of **4**-*rac* with (*R*)-mandelic acid afforded the desired amine enantiomer (*S*)-**4**-mandelate (Scheme 1a).^8,11^ Gilead also prepared the enantiopure amine fragment from the reaction of 3,6-dibromopicolinaldehyde (**1**) with (*S*)-*tert*-butylsulfinamide. The resulting chiral imine provides the desired amine enantiomer (*S*)-**4** (as an HCl salt) after the addition and hydrolysis steps.^9,10^ Recently, Lipshutz and co-workers disclosed an efficient route to the ketone intermediate **8** (Scheme 1b).^12^ The sequence involves the reaction of aldehyde **1** with the organozinc reagent to provide the alcohol intermediate (**7**). Later, Turner’s group transformed the ketone **8** to chiral amine (*S*)-**4** by an engineered aminotransferase.^13^ Several other chemical transformations of ketone **8** to access chiral amine (*S*)-**4** with transition metal-catalyzed hydrogenation have been disclosed by Gilead (Scheme 2).^8^ For instance, the asymmetric hydrogenation of vinyl acetamide using a chiral iridium catalyst afforded (*S*)-**4**-Ac. Reductive amination of ketone **8** with a chiral ruthenium catalyst yielded (*S*)-**4**. Ketone **8** could be reduced to a chiral alcohol with a chiral ruthenium catalyst and then converted to (*S*)-**4**-HCl under Staudinger reaction conditions.

Among these reported methods for synthesis of the enantiopure amine fragment, the resolution approach (Scheme 1a) is particularly attractive because this methodology eliminates the need for expensive enzymes or transition metal catalysts and ligands. Moreover, dynamic kinetic resolution (DKR) has emerged as an important tool in asymmetric synthesis, with a theoretical yield of 100% rather than 50% from the classical resolution approach.^14,15^ Given the anticipated need for large-scale lenacapavir synthesis, we have sought a scalable synthetic route to the enantiopure amine fragment based on the DKR chiral resolution approach. Herein, we describe a novel seven-step sequence utilizing DKR for the synthesis of the enantiopure amine fragment (*S*)-**4**, which requires no chromatographic purification steps.^16^

## Results and Discussion

The synthesis of ketone **8** is imperative to the success of the chemistry to access amine **4**-*rac*. Our initial efforts to make ketone **8** involved the nucleophilic substitution of 3,5-difluorobenzylnitrile **9a** with organomagnesium reagent **10**-Mg (Table 1). Organomagnesium reagent **10**-Mg was prepared by deprotonation of **10** with Knochel-Hauser base (TMPMgCl·LiCl), according to the reported protocol.^8^ Treatment of **10**-Mg with **9a** failed to generate any desired product (Table 1, Entry 1). Efforts to access ketone **8** by activating **9a** for **10**-Mg addition with ZnCl_2_ were not successful (Table 1, Entry 2). Switching the organomagnesium reagent to organolithium salt **10**-Li also failed to afford ketone **8**; partially scrambled lithiation and debromination were observed. With a decrease in the reaction temperature or the addition of metal chelating agents, such as HMPA and TMEDA, no improvement was observed. All these attempts resulted in mainly the recovery of **9** or **10** (Table 1, Entries 3–4). We postulated that the organometallic reagent **10**-Mg or **10**-Li was quenched by the acidic benzylic C–H bonds of **9a** (p*K*_a_ ∼ 19–20)^17^ negating the desired 1,2-addition of the nitrile.

**Table 1:**
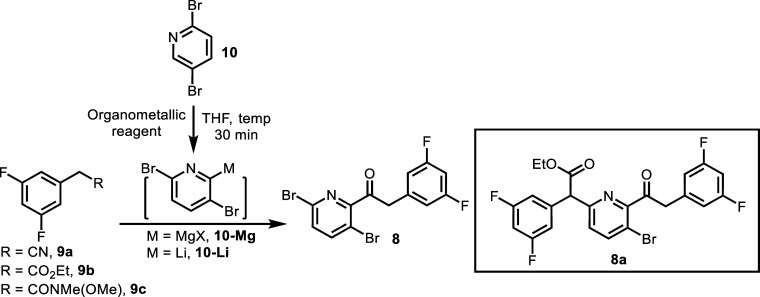
Synthesis of Ketone **8**

entry^a^	organometallic reagent (eq)	electrophile (eq)	temp (°C)	GC-MS analysis(TIC A%)^b^		
				8	9a/9b/9c	10
1	TMPMgCl·LiCl (1.5)	**9a** (1.5)	–20	ND	50 (**9a**)	45
2	TMPMgCl·LiCl (2.5)/ZnCl_2_ (1.1)	**9a** (1.1)	–20	ND	30 (**9a**)	60
3^c^	LDA (1.0)/HMPA (10%)	**9a** (1.2)	–40	ND	57 (**9a**)	2
4	LiHMDS (1.0)/HMPA (10%)	**9a** (1.2)	–40	ND	31 (**9a**)	50
5^d^	TMPMgCl·LiCl (1.5)	**9b** (1.5)	–20	20	31 (**9b**)	30
6	TMPMgCl·LiCl (1.1)	**9c** (1.1)	–10	51		40
7	TMPMgCl·LiCl (1.0)	**9c** (1.1)	–20	67		25
8	TMPMgCl·LiCl (1.1)	**9c** (1.1)	–20	85		10
9^e^	TMPMgCl·LiCl (1.1)	**9c** (1.1)	–20	68^f^	5 (**9c**)	10

a All reactions were carried out with **10** (0.25 g, 1 equiv) and organometallic reagent in THF (10 V) for 30 min, followed by addition of electrophile and stirring at the same temperature for 2 h under the condition shown in the table unless otherwise stated, solvent volume (V) = mL/g of **10**.

b All these data were in-process analysis data of crude reaction mixtures by GC-MS total ion chromatogram (TIC) and reported as Area% (A%) unless otherwise stated.

c A possible *N*,*N*-diisopropylpyridin-2-amine (42 A%, *m*/*z* 179) was observed by GCMS.

d 4 h, 20A% of adduct **8a** from the reaction of **9b** with **8** was observed.

e Reaction was conducted on 30 g scale.

f Extractive workup and subsequential trituration for purification, 50% yield of the product was obtained with 93% purity (qNMR) after trituration from 5% ethyl acetate in heptanes. ND: the desired product was not detected.

We envisioned that a decrease in acidity of the benzylic C–H bonds of the electrophile might mitigate deprotonation and thus favor the addition reaction. In this regard, the reaction of **10**-Mg with ester **9b** (p*K*_a_ ∼ 22–23)^17^ afforded the desired product in 20 A% (Table 1, Entry 5). The positive result inspired the investigation of other electrophiles. Weinreb amide **9c** (p*K*_a_ ∼ 26) was identified as the optimal electrophile to afford ketone **8**. The reaction of **10**-Mg with Weinreb amide **9c** afforded ketone **8** in 51 A% (Table 1, Entry 6). We posit that the cumulative factors, less acidic benzylic C–H bonds, and exceptional metal chelation capability of the Weinreb amide facilitate ketone formation.^18^ Anhydrous THF was the optimal solvent for this transformation. Further optimization of equivalents of Knochel–Hauser base and reaction temperature revealed that 1.1 eq of Knochel-Hauser base (TMPMgCl·LiCl) and −20 °C delivered the best result of ketone **8** (Table 1, Entries 7–8). Under these best conditions, the reaction of **10**-Mg with Weinreb amide **9c** afforded ketone **8** in 85 A% and 66% isolated yield was obtained after column chromatography purification (Table 1, Entry 8). To eliminate column purification, a process of an extractive workup and subsequent trituration was identified to allow the isolation of **8**. The protocol was demonstrated on a 30g scale, afforded **8** in 50% isolated yield with 93% purity (qNMR) (Table 1, Entry 9). Weinreb amide **9c** was readily synthesized from commercially available 2-(3,5-difluorophenyl)acetic acid (**9**)^19^ in a one-pot process. Conversion of 2-(3,5-difluorophenyl)acetic acid to acetyl chloride followed by reaction with N,O-dimethylhydroxyl-amine afforded Weinreb amide **9c** in 96% isolated yield (see the SI for experimental details).

**Scheme 3:**
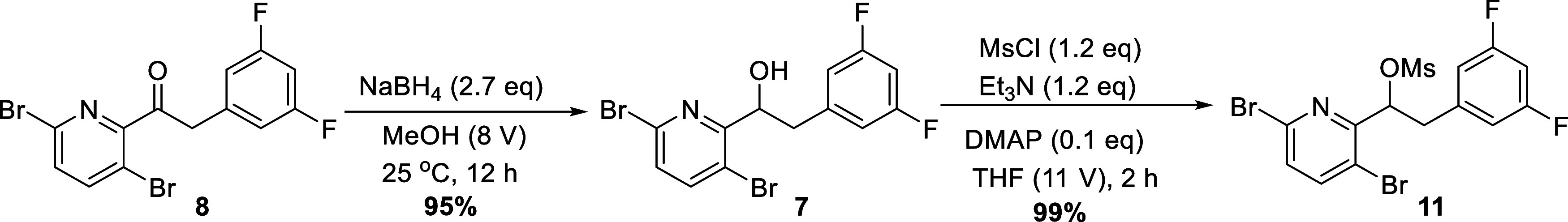
Synthesis of Mesylate **11** from Ketone **8**

With ketone **8** in hand, our effort was focused on the synthesis of the racemic amine (**4**-*rac*). Initial attempts to achieve **4**-*rac* were performed by Leuckart amination of **8** with NH_4_HCO_2_,^20^ but this resulted in the recovery of the starting material **8**. Other amines, such as Boc-NH_2_^21^ and *t*-BuS(O)NH_2_^22^ also failed to react with ketone **8**. Ultimately, the reduction of ketone **8**, mesylation of the incipient alcohol, and then amination of mesylate **11** successfully afforded **4**-*rac* (Table 2). Mesylate **11** was prepared in two steps from ketone **8** in >95% isolated yield (Scheme 3). Reduction of ketone **8** with NaBH_4_ proceeded smoothly^23^ and afforded alcohol **7** in >95% isolated yield (95% qNMR purity). The subsequent mesylation of alcohol **7** with mesyl chloride furnished **11** in >99% isolated yield (93% qNMR purity).

**Table 2:**
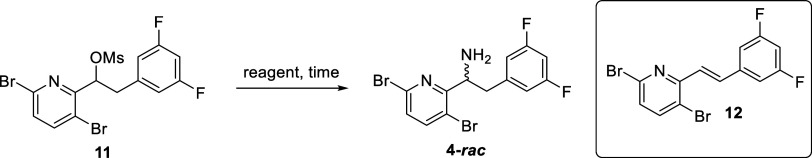
Optimization of Amination **11** for the Synthesis of **4**-*rac*

entry^a^	reagents (20 V)	time (h)	isolated yield (%)
1	aq. 30% NH_4_OH	24	48^b^
2	7 M NH_3_ in MeOH	24	46^b^
3	7 M NH_3_ in MeOH	40	56^c^
4	7 M NH_3_ in MeOH	18	NR^d^
5	aq. 30% NH_4_OH	18	NR^d^
6	aq. 30% NH_4_OH	24	54^e^

a All reactions were carried out at 70 °C with 20 volumes of aminating solution as shown in the table, volume = mL/g of **11**.

b The reaction was performed on 100 mg of **11** in a heavy wall reactor; purification by column chromatography.

c The reaction was performed with **11** (10 g, 20.7 mmol) and 7 M NH_3_ in MeOH (200 mL) in a Parr reactor; the inside pressure was recorded as 49 psi; the product was purified with column chromatography; olefin **12** was isolated in 22% yield.

d Reaction was performed in conventional glass reactors, at ambient pressure, NR: no reaction occurred.

e Reaction was performed with **11** (10 g, 20.7 mmol), 30% aq. NH_4_OH (200 mL) and MeOH (40 mL) in a Parr reactor; the inside pressure was recorded as 50 psi; the product was purified with an aqueous pH-attuned workup process.

The treatment of **11** with NH_4_OH in a heavy wall pressure vessel at 70 °C afforded **4**-*rac* in 48% isolated yield with 99A% purity (GC-MS) (Table 2, Entry 1). Similarly, conducting the amination with a solution of NH_3_ in MeOH provided **4**-*rac* in 46% isolated yield with 99A% purity (GC-MS) (Table 2, Entry 2). The amination with NH_3_ in MeOH scaled up to a 10 g scale, afforded **4**-*rac* in 56% isolated yield (Table 2, Entry 3). Notably, 40 h of reaction time was needed to achieve a full conversion in this reaction. Under these conditions, the mesylate elimination product, olefin **12**, was concurrently formed. Conducting experiments in conventional glass reactors at ambient pressure resulted in no reaction (Table 2, Entries 4–5). Parenthetically, the purification of **4**-*rac* was accomplished by column chromatography. To avoid column chromatography a practical aqueous pH-attuned workup process was developed to make a pure **4**-*rac*. The protocol was demonstrated on a 10 g scale with NH_4_OH as an aminating reagent. For example, after completion of the amination in a Parr reactor, the reaction mixture was treated with aq. HCl. This acid treatment allows complete elimination of side product **12** by ethyl acetate extraction, leaving **4**-*rac*-HCl in the aqueous phase. Adjusting the **4**-*rac*-HCl enriched aqueous layer to an alkaline pH with aq. NaOH afforded pure **4**-*rac* as a solid, after crystallization in 54% isolated yield with 99% purity by qNMR (Table 2, Entry 6).

With the racemic amine **4**-*rac* in hand, our efforts were initially focused on kinetic resolution. A variety of chiral acids were screened as resolving reagents. As summarized in Table 3, (*R*)-(−)-mandelic acid was found to be the best-resolving agent, affording the enantiomer in up to 31% isolated yield and 100% *de* (Table 3, Entries 1–3). Other resolving agents, such as *L*-(+)-tartaric acid, N-acetyl-d-leucine (NADL), and (*1R*)-(−)-10-camphorsulfonic acid failed to yield the resolute in high diastereomeric excess (Table 3, Entries 4–6).

**Table 3:**
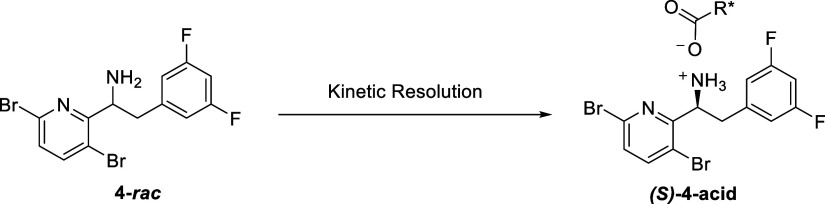
Kinetic Resolution of **4**-*rac* To Access Chiral Amine

entry^a^	resolving agent	crystallization condition	(*S*)*-*4-acid	*de* (%)^b^	isolated yield (%)	[α]_*D*_^20^
1	(*R*)-mandelic acid	0 °C, 1–2 h	(*S*)*-***4**-mandelate	98.6	22	
2	(*R*)-mandelic acid	–10 °C, 1–2 h	(*S*)-**4**-mandelate	100	23	
3^c^	(*R*)-mandelic acid	0 °C, 17 h	(*S*)-**4**-mandelate	100	31	+31.22^d^
4	*L*-(+)-tartaric acid	–10 °C, 1–2 h	(*S*)-**4**-tart	37.8	80	
5	N-acetyl-d-leucine	–10 °C, 1–2 h	(*S*)*-***4**-NADL	9.6	43	
6	(1*R*)-(−)-10-camphorsulfonic acid	–10 °C, 1–2 h	(*S*)*-***4**-camp	2.8	94	

a All resolutions were carried out with **4**-*rac* (1 g, 1 equiv) and chiral acid (1 equiv) in MTBE/toluene (7/3, v/v, 10 V), 60 °C for 2–4 h, solvent volume (V) = mL/g of **4**-*rac*.

b Diastereomeric excess (*de*) was measured by SFC (210 nm).

c Attempts to improve yield with (*R*)-mandelic acid failed. Recrystallization at 25 °C resulted in no solid precipitates. A longer recrystallization time (65 h) slightly increased yield (36%), but de dropped to 96% *de*.

d Optical rotation number was measured in MeOH (10 mg/mL) at 20 °C under 589 nm, the number shown in the table is the specific optical rotation number (deg·mL·g^–1^·dm^–1^).

**Scheme 4:**
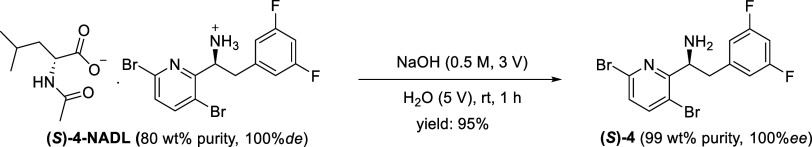
Synthesis of Enantiopure Free Amine (*S*)-**4**

**Figure 2:**
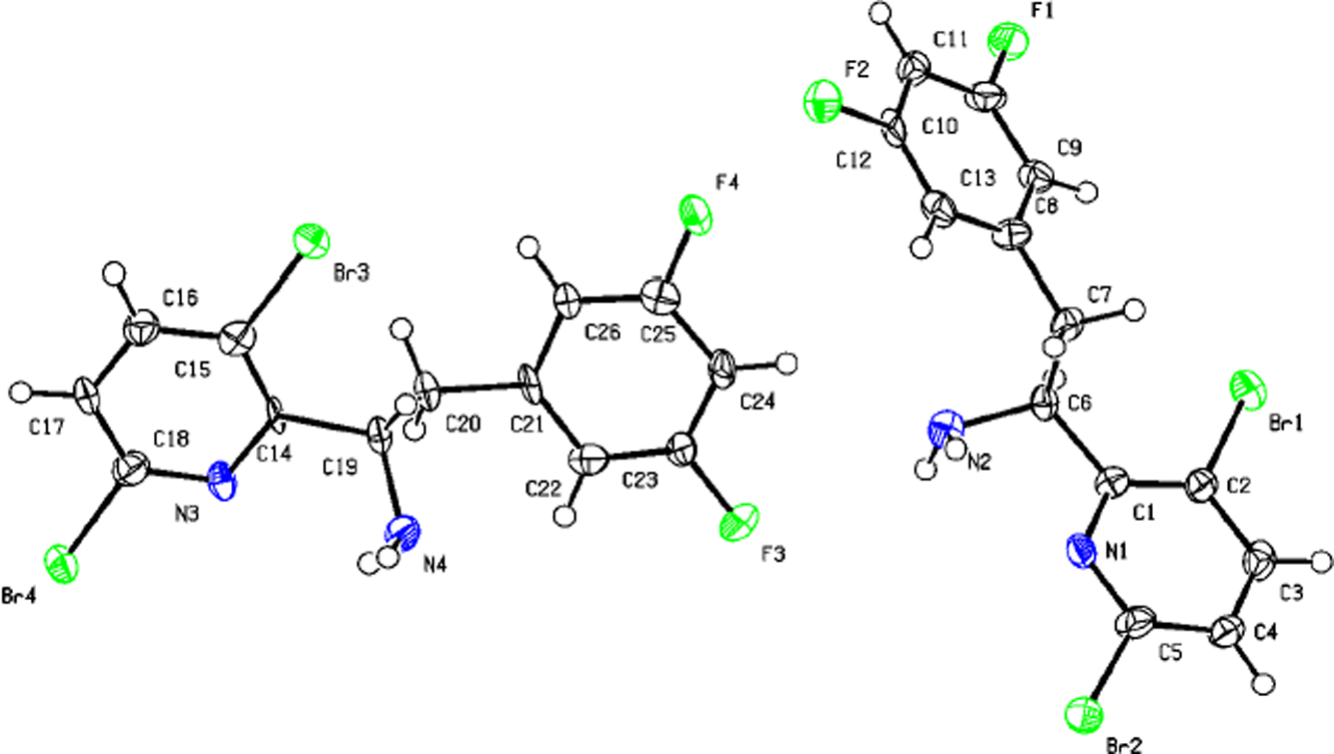
X-ray structure of (*S*)-**4** (confirmed (*S*)-absolute configuration of the chiral amine center).

With these results, our efforts shifted to dynamic kinetic resolution (DKR). Also known as crystallization-induced stereoisomer transformation, DKR has emerged as an important tool in asymmetric synthesis.^14,15^ Most importantly, the DKR of a racemate allows for a theoretical yield of 100% rather than 50% from a classical resolution approach. Using N-acetyl-d-leucine (NADL) to resolve **4**-*rac* was disclosed by Gilead.^24,25^ To our delight, the resolution of **4**-*rac* with NADL in the presence of 5 mol % pyridine-2-carboxaldehyde and 10 mol % ZnO afforded (*S*)-**4**-NADL in 61% yield with excellent diastereoselectivity (99.6% *de*) (Table 4, Entry 1). Replacement with inexpensive (*R*)-(−)-mandelic acid as the resolving agent, the resolution afforded (*S*)-**4**-mandelate in a much lower isolated yield (28%) but excellent enantioselectivity (100% *de*) (Table 4, Entry 2). Thus, the reaction with NADL was repeated in a 10g-scale of **4**-*rac* (Table 4, Entry 3). After resolution, the (*S*)-**4**-NADL salt was obtained in 63% corrected overall yield with 100% *de*. Notably, the wt % of the obtained salt was 80%, and analysis of the NMR spectra showed the presence of approximately 20 wt % of free NADL. To remove NADL, the salt was treated with aq. NaOH (1.3 eq, 1M). This treatment removed the NADL completely, and free amine (*S*)-**4** was obtained in 95% yield with 99% qNMR purity and 100% *ee* (Scheme 4). The X-ray structure of (*S*)-**4** obtained by single crystal X-ray crystallography confirmed the (*S*)-absolute configuration of the enantiopure amine (Figure 2).

**Table 4:**
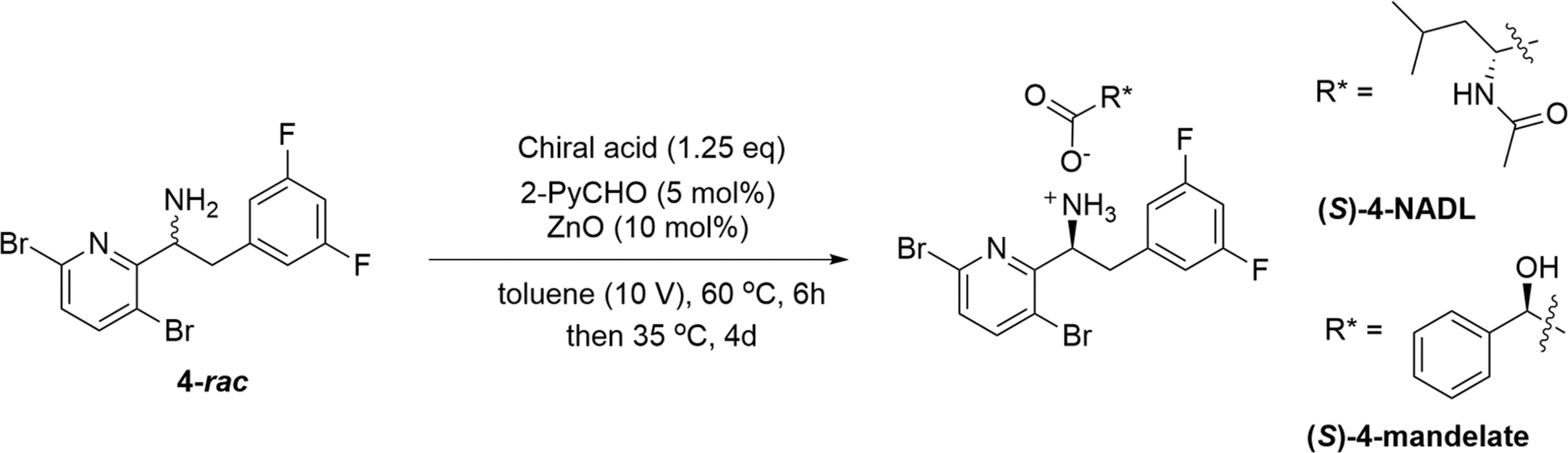
Chiral Acids Screen for DKR of **4**-*rac*

entry	scale	chiral acid	product (output)	*de* (%)^c^	yield (%)^d^	wt %	[α]_*D*_^20^^e^
1^a^	1 g	N-acetyl-d-Leucine	(*S*)-**4**-NADL (1.2 g)	99.6	61	73^f^	+66.1
2^b^	1 g	(*R*)-mandelic acid	(*S*)-**4**-mandelate (0.4 g)	100	28	60^f^	+31.2
3	10 g	N-acetyl-d-Leucine	(*S*)-**4**-NADL (11.5 g)	100	63	79^g^	+64.4

a Dynamic Kinetic Resolution of **4**-*rac* with N-acetyl-d-leucine (1.25 equiv), 2-PyCHO (5 mol %), ZnO (10 mol %), toluene (10 V), 60 °C, 6h, then 35 °C, 4 days.

b Dynamic kinetic resolution of **4**-*rac* with (*R)*-(−)-mandelic acid (1.25 equiv), 2-PyCHO (5 mol %), ZnO (10 mol %), toluene (10 V), 60 °C, 6h, then 35 °C, 4 days.

c The diastereomeric excess (de) was measured by SFC (210 nm).

d Corrected isolated yield based on wt % purity.

e Specific rotation was recorded in MeOH (10 mg/mL) at 20 °C under 589 nm.

f wt % was obtained by qNMR.

g wt % was obtained by HPLC (210 nm).

**Scheme 5:**
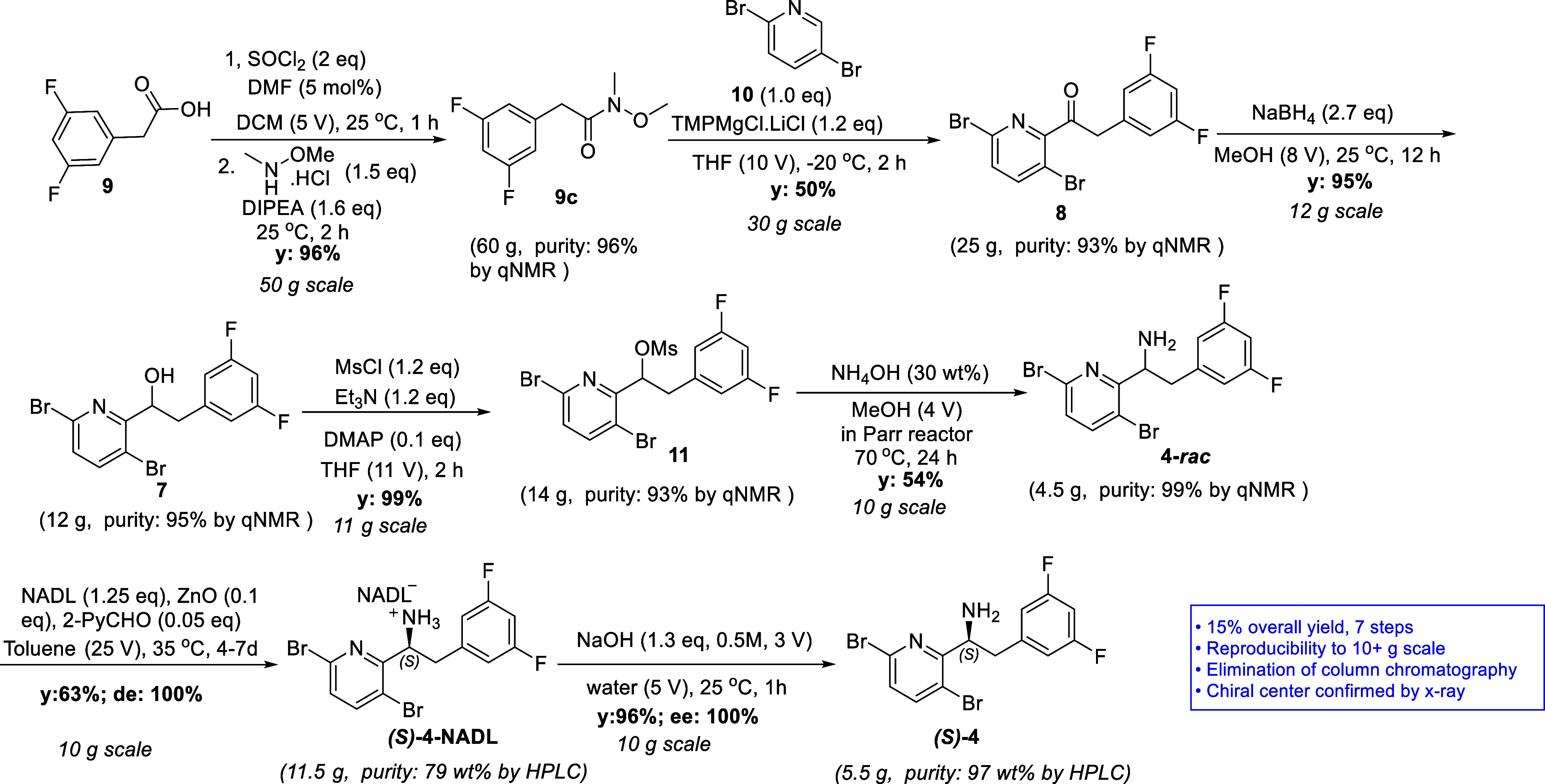
Decagram-Scale Demonstration of Synthesis of Chiral Amine (*S*)-**4**

To further demonstrate the synthetic utility of our 7-step protocol for the preparation of (*S*)-**4**, decagram-scale batches were carried out (Scheme 5). Starting with 50 g of 2-(3,5-difluorophenyl)acetic acid (**9**), the Weinreb amide **9c** was isolated with 95% yield. Treatment of the Weinreb amide **9c** with 2,5-dibromopyridine (**10**) in the presence of TMPMgCl furnished the ketone **8** in 50% isolated yield with >99% qNMR purity. The ketone **8** was then reduced with NaBH_4_ to afford alcohol **7** in a 95% isolated yield. Mesylate **11** was obtained in almost quantitative yield by the mesylation of alcohol **7**. Amination of mesylate **11** with NH_4_OH in a Parr reactor delivered **4**-*rac* in 54% isolated yield with >99% qNMR purity after an acid–base treatment. The obtained **4**-*rac* was resolved with NADL to afford (*S*)-**4**-NADL in 66–68% isolated yield with 76–80 wt % purity (HPLC) and 100% *de*. After treating with aq NaOH, (*S*)-**4** was obtained as a white solid in 96% yield with 97 wt % purity and 100% *ee*. As a result, the overall isolated yield of (*S*)-**4** from 2-(3,5-difluorophenyl)acetic acid (**9**) was 15–16% with this methodology. It is noteworthy that this synthetic route does not require chromatographic purifications.

## Conclusions

In conclusion, a new synthetic route has been disclosed to access the enantiopure amine (*S*)-**4**, a fragment needed for the synthesis of lenacapavir. This strategy features Weinreb amide ketone synthesis, nucleophilic amination, and dynamic kinetic resolution as key steps. This route utilizes readily available and inexpensive raw materials and reagents, avoiding transition metal catalysts and costly enzymes. As a synthetic method without chromatographic purifications, it is amenable for scale-up and should improve access to the chiral amine (*S*)-**4** for the manufacture of the anti-HIV drug lenacapavir.

## Data Availability

The data underlying this study are openly available in the published article and its Supporting Information.

## Supporting Information Available

Additional experimental details and analytical methods (PDF)
