# Supplementary material for: A New Synthesis of Enantiopure Amine Fragment: An Important Intermediate to the Anti-HIV Drug Lenacapavir
Source: J Org Chem. 2024 Dec 16;90(1):471–8. doi: 10.1021/acs.joc.4c02380 (PMC11731271; doi:10.1021/acs.joc.4c02380)
Supplement: Supplementary file 1 — jo4c02380_si_001.pdf [file jo4c02380_si_001.pdf]

Supporting Information

A new synthesis of enantiopure amine fragment: an important intermediate to the anti-HIV drug lenacapavir

*Anand H. Shinde,[‡] Ramakrishna Sayini,[‡] Piyal Singh,[‡] Justina M. Burns, Saeed Ahmad, G. Michael Laidlaw, B. Frank Gupton, Douglas Klumpp, Limei Jin**

Medicines for All Institute, Virginia Commonwealth University, Richmond, VA, 23284-3068.

[‡]These authors contributed equally.

*Corresponding author: Email: jinl3@vcu.edu

Table of Contents

General Method S-2

GC-MS Method S-2

HPLC-UV Method S-5

Experimental procedure S-9

NMR Spectra and X-ray data S-18

General Method

Reagents and solvents were obtained from commercial suppliers and used as received unless otherwise indicated. Where applicable, reactions were conducted in oven-dried (120˚C) glassware, which was assembled while hot, and cooled to ambient temperature under an inert atmosphere. Reactors were pre-rinsed with reaction solvent and subjected to evacuation/back-fill cycles (3×) as necessary. Reactions were monitored by TLC (precoated silica gel 60 F254 plates, EMD Chemicals), Agilent GCMS, Agilent LCUV or crude ^{1}H NMR. TLC was visualized with UV light. The proton (^{1}H NMR), carbon (^{13}C NMR) and 2-DNMR spectra of the compounds were recorded on Bruker Avance III HD Ascend 600 MHz spectrometer. The NMR solvents used were DMSO-d_6 and $CDCl_3$. The chemical shifts were reported in parts per million (ppm). Coupling constants J are reported in hertz (Hz). The abbreviations used to designate signal multiplicity were: s, singlet; d, doublet; t, triplet; q, quartet, p, pentet; dd, doublet of doublets; ddd, doublet of doublet of doublets; dt, double of triplets; ddt, doublet of doublet of triplets; m, multiplet; br, broad. 1,3,5-trimethoxybenzene and/or triphenylmethane, were used as internal standards for quantitative ^{1}H-NMR.

GC-MS Method

The formation of all compounds was monitored via GC-MS (Agilent 8890 GC-5977 MSD). An Agilent J&W HP-5ms GC Column, 30 m, 0.25 mm, 0.25 µm, 7-inch cage was used for analysis. The inlet was set to 250 ˚C. A split ratio of 100:1 was used with an injection volume of 1.0 µL. The column flow rate was 1.4 mL/min with ultra-high purity helium as the carrier gas and an inlet pressure of 7.87 psi. The oven was initially set to 50 ˚C for 3 minutes, linearly ramped to 250 ˚C at 25 ˚C/min, and held for 3 minutes. The column temperature was ramped a final time at 25 ˚C/min to 300 ˚C which was held for 3 min.

Structures & IDs:

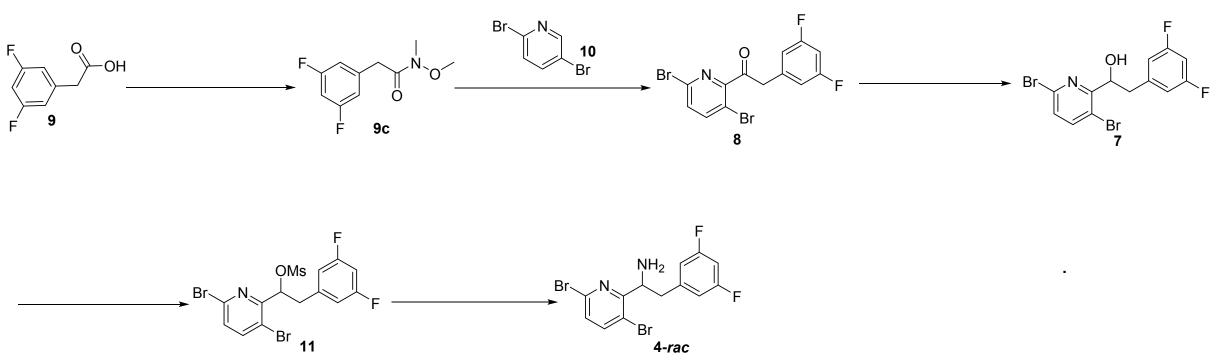

Instrument Type: Agilent 8890 gas chromatograph (GC) with a 5977 mass spectrometer detector (MSD)

Conditions:

Column: J&W HP-5ms GC Column, 30 m, 0.25 mm, 0.25 μm, 7 inch cage

Inlet Pressure: 11.747 psi | Split Ratio: 100:1 | Split Flow: 140 mL/min

Column flow: 1.4 mL/min | Injection Temp: 250°C | Injection volume: 1 μL

Total Flow: 144.4 mL/min | Solvent Delay: 3 min | Runtime: 19 min

Temperature Program:

Time (min)	Temp (°C)	Ramp (°C/min)	Hold (min)
0	50	-	3
-	25	250	3
-	25	300	3

MS Parameters:

Transfer Line Temp (°C)	250
Source Temp (°C)	230
Quad Temp (°C)	150
Electron Energy (eV)	70
Mass Range	40-1000

Sample preparation: Samples are prepared at ~1 mg/mL in acetonitrile

Retention Times

Compound	Primary *m/z*	Secondary *m/z*	Time (min)
**9**	127	200	7.5
**10**	237	156	7.8
**9c**	127	215	8.8
**8**	264	391	12.2
Dimer of **10**	472	312	14.4
Double addition of **8**	511	202	16.5

Notes:

Representative Chromatogram(s) (attach additional chromatograms and spectra as needed)

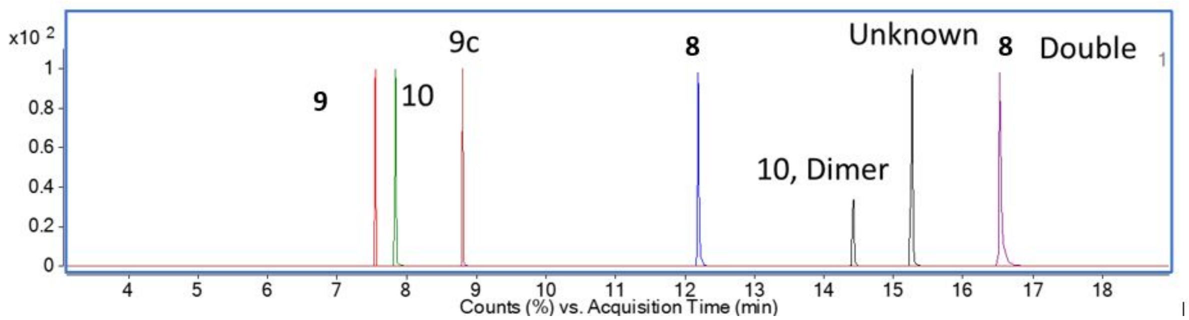

Mass spectra:

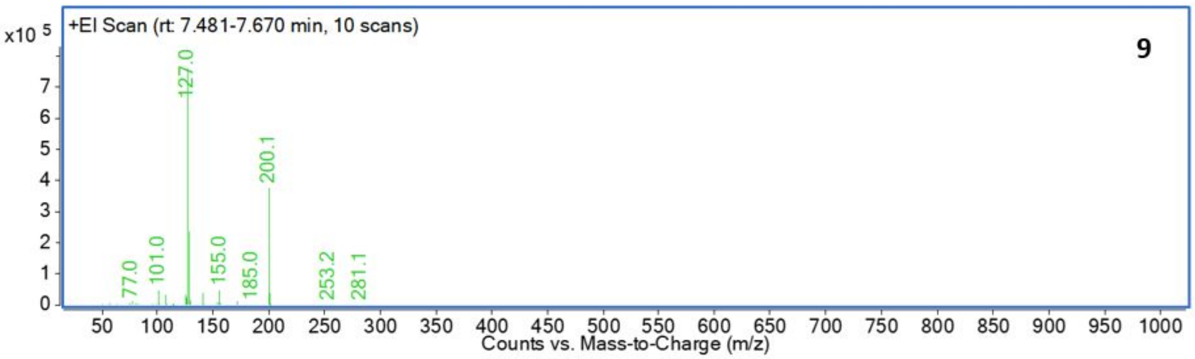
x10 5
+EI Scan (rt: 7.481-7.670 min, 10 scans)
9
127.0
200.1
77.0
101.0
155.0
185.0
253.2
281.1
Counts vs. Mass-to-Charge (m/z)

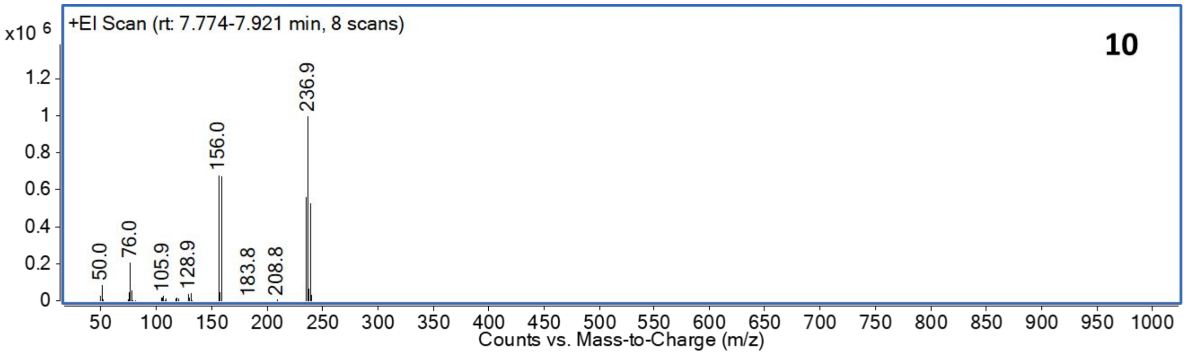
x10 6
+EI Scan (rt: 7.774-7.921 min, 8 scans)
10
236.9
156.0
50.0
76.0
105.9
128.9
183.8
208.8
Counts vs. Mass-to-Charge (m/z)

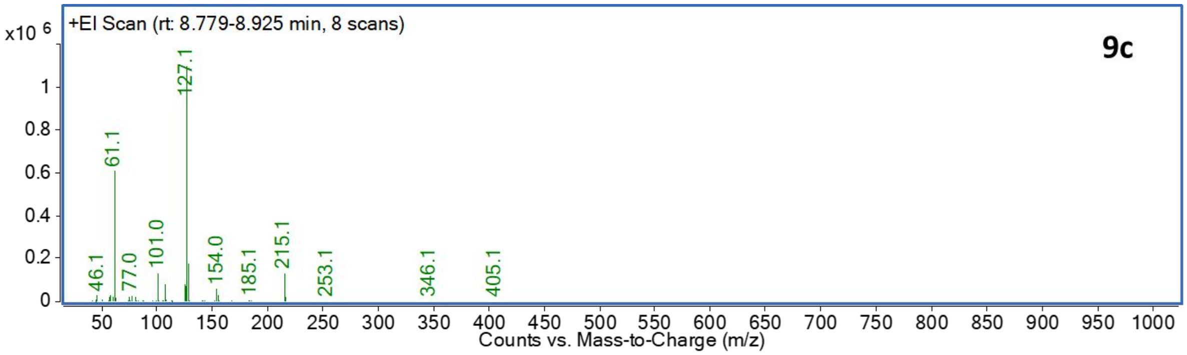
x10 6
+EI Scan (rt: 8.779-8.925 min, 8 scans)
9c
127.1
61.1
46.1
77.0
101.0
154.0
185.1
215.1
253.1
346.1
405.1
Counts vs. Mass-to-Charge (m/z)

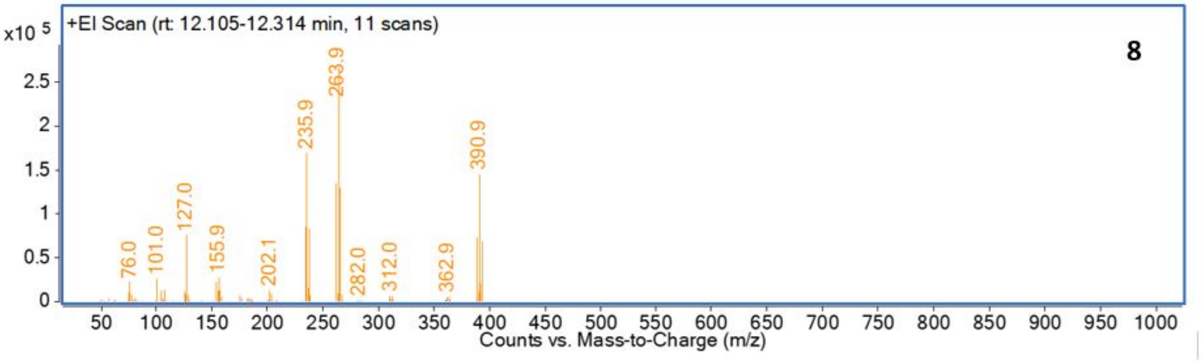
x10 5
+EI Scan (rt: 12.105-12.314 min, 11 scans)
8
263.9
235.9
390.9
76.0
101.0
127.0
155.9
202.1
282.0
312.0
362.9
Counts vs. Mass-to-Charge (m/z)

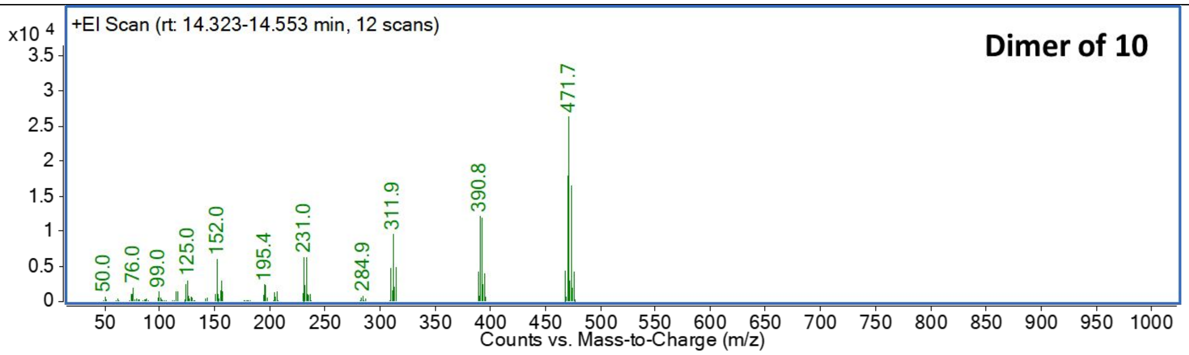

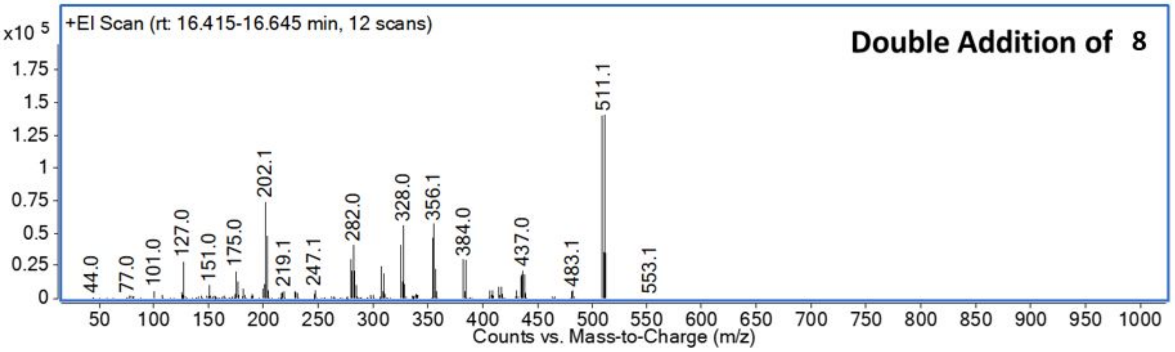

HPLC-UV Method

Formation of intermediates through the product **4** was monitored via LC-UV (Agilent 1100 LC-diode array detector). An Agilent ZORBAX RR Eclipse Plus C18, 4.6 x 100 mm, 3.5 µm was used for analysis. The column temperature was set to 30 ˚C. Mobile phase A was 0.1% phosphoric acid in 18 megohm water and mobile phase B was acetonitrile. The column flow rate was 1.5 mL/min with initial conditions being set to 40% B. This was held for 0.5 min and then ramp to 95% B over 4.5 min and held for 5 min. A 2 min post time was used. The detector was set to 210 nm.

Structures & IDs:

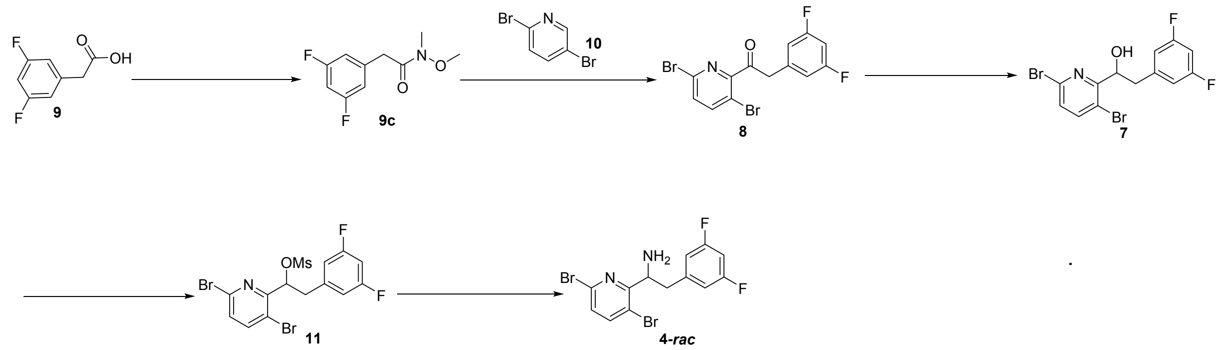

Instrument Type: Agilent 1100 liquid chromatograph (LC) with diode array detector (DAD)

Conditions:

Column: Agilent ZORBAX RR Eclipse Plus C18, 4.6 x 100 mm, 3.5 µm
Mobile Phase A: 0.1% phosphoric acid in water
Mobile Phase B: Acetonitrile
Injection volume: 1 µL   Column temp: 30°C   Flow rate: 1.5 mL/min
Detector wavelength(s): 210 nm

LC Gradient Table:

Time (min)	%A	%B
0	60	40
0.5	60	40
5	5	95
10	5	95

Post-run equilibration: 2 minutes

Sample preparation: Prepare samples at 1 mg/mL in acetonitrile

Retention Times			
**Compound**	**Time (min)**	**Relative RF (mg/mL)***	**Relative RF (M)***
**4**	0.95		
**9**	1.6		
**10**	3.0		
**7**	4.3		
**11**	4.3		
**8**	4.8		

Notes:

$$*Relative\ RF = \frac{\left({}^{Analyte\ 2\ Conc.}/_{Analyte\ 2\ Peak\ Area}\right)}{\left({}^{Analyte\ 1\ Conc.}/_{Analyte\ 1\ Peak\ Area}\right)}$$

Representative Chromatogram(s) (attach additional chromatograms and spectra as needed)

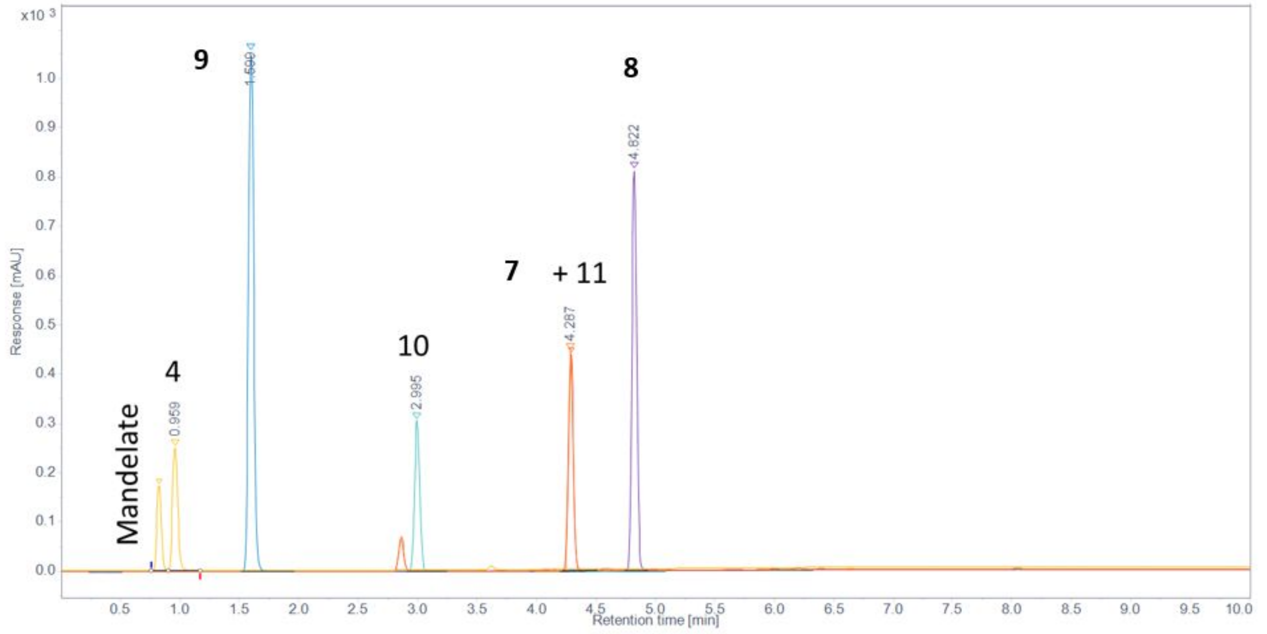

UV Spectra

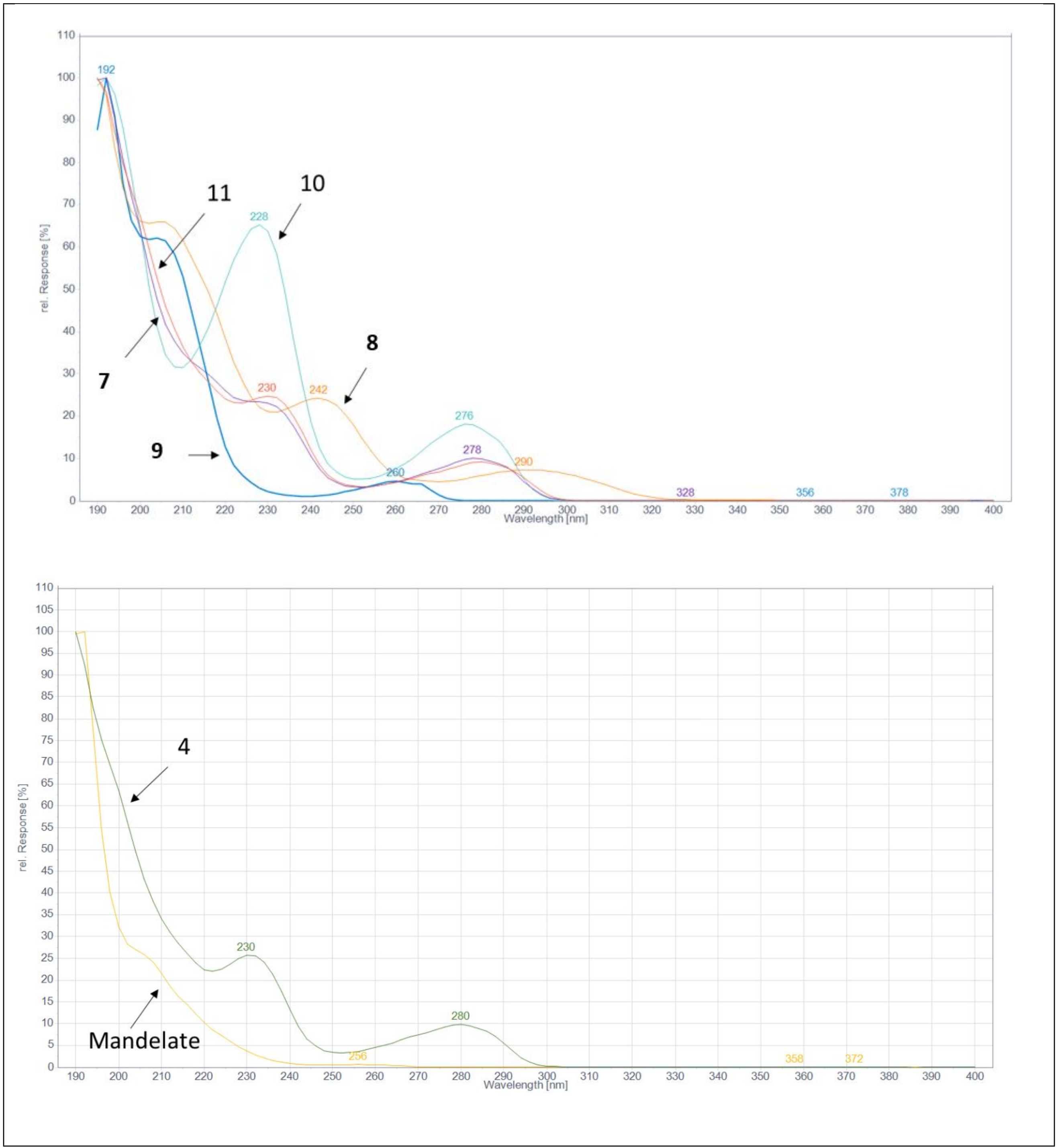

SFC Method

Structures & IDs:

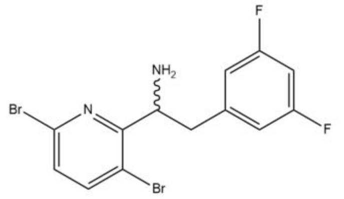

1-(3,6-dibromopyridin-2-yl)-2-(3,5-difluorophenyl)ethan-1-amine
Exact Mass: 389.92

Instrument Type: Agilent 1260 Infinity super critical fluid chromatograph (SFC) with diode array detector (DAD)

Conditions:

Column: Chiral Technologies CHIRALPAK IA SFC, 4.6 x 250 mm, 3 µm

Mobile Phase A: CO_2

Mobile Phase B: Methanol

Injection volume: 5 µL Column temp: 25˚C Flow rate: 2.0 mL/min

BPR Pressure: 100 bar BPR temp: 60˚C

Detector wavelength(s): 210 nm

Gradient Table:

Time (min)	%A	%B
0	90	10
10	90	10

Sample preparation: Prepare samples at 1 mg/mL in acetonitrile

Post-run equilibration: NA

Retention Times			
**Compound**	**Time (min)**	**Relative RF (mg/mL)***	**Relative RF (M)***
(R)-4	4.2	-	-
(S)-4	6.8	-	-

Representative Chromatogram

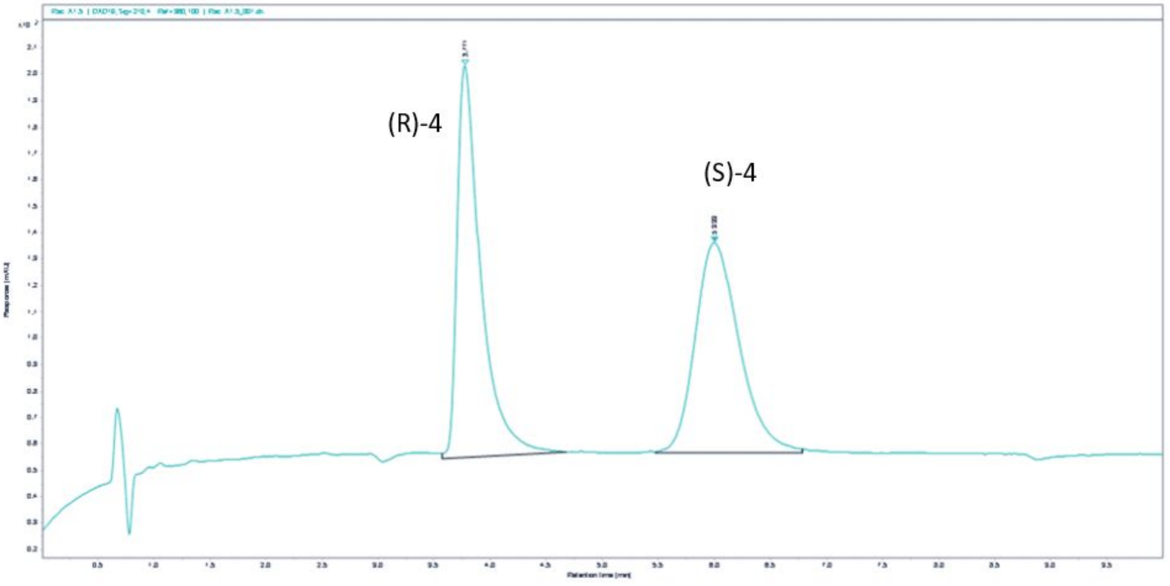

UV Spectra

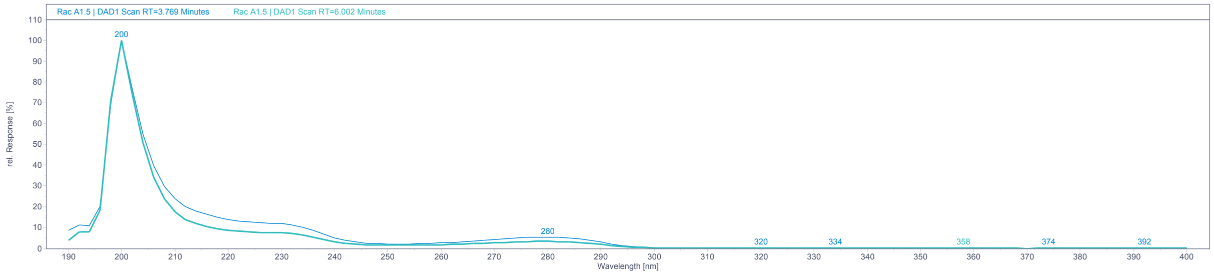

Experimental procedure

2-(3,5-difluorophenyl)-N-methoxy-N-methylacetamide (9c)

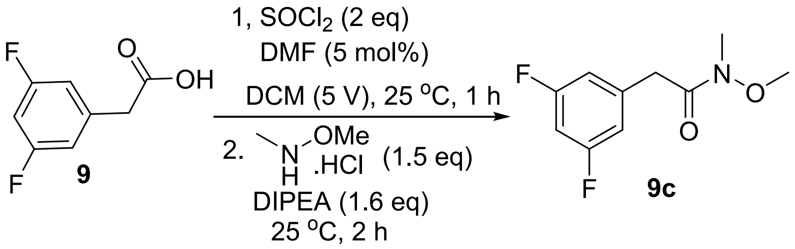

To a 1L three-necked round flask equipped with an overhead stirrer was added 2-(3,5-difluorophenyl) acetic acid (**9**, 50.0 g, 290.48 mmol, 1 eq), DMF (1.06 g, 1.12 mL, 14.5 mmol, 0.05 eq) and dichloromethane (250 mL). The mixture was stirred for 10 min under N_2 atmosphere

and cooled 0-5 °C. To the mixture was added $SOCl_2$ (69.11 g, 59.42 mL,580.96 mmol, 2 eq) slowly. After addition, the mixture was stirred at 25 °C for 1 h. After completion of the reaction, the volatiles were removed under vacuum and further evaporated to dryness to afford the crude acid chloride as a yellow viscous oil (TIC Area % 97.49 by GCMS). The obtained oil was dissolved in dichloromethane (40 mL) for the next step.

In another oven-dried 1L, three-necked round flask equipped with an overhead stirrer was charged N,O-dimethyl hydroxylamine hydrochloride (42.50 g, 435.72 mmol, 1.5 eq) and DCM (200 mL). The mixture was stirred for 10 min and cooled to 0 °C. DIPEA (60.07 g, 81.0 mL, 435.72 mmol, 1.6 eq) was added dropwise. After addition, the mixture was stirred at 0 °C for 30 min. The above solution of acid chloride in DCM (40 mL) was added dropwise and the resulting solution was stirred at 25 °C overnight. After completion of the reaction, the mixture was cooled to 0-5 °C, quenched with 0.2 M HCl (100 mL), and extracted with DCM (2 × 250 mL). The combined organic layer was washed with brine and evaporated to dryness to afford sticky light yellow solid **9c** (60 g, 96 % isolated yield, 98.3% purity by GCMS, 96% purity by qNMR with 1,3,5-trimethoxybenzene as internal standard).

^1H NMR (600 MHz, $CDCl_3$): δ 6.88 – 6.76 (m, 1H), 6.72 – 6.63 (m, 2H), 3.72 (s, 3H), 3.64 (s, 2H), 3.18 (s, 3H).

$^{13}C\{^1H\}$ NMR (151 MHz, $CDCl_3$): δ 171.1, 163.0 (dd, J = 248.1, 12.9 Hz), 138.6, 112.5 (dd, J = 20.2, 5.1 Hz), 102.5 (t, J = 25.3 Hz), 61.5, 38.9, 32.4.

^{19}F NMR (565 MHz, $CDCl_3$) δ -110.3 (s).

IR (ATR, DCM) ν_{max} = 2950, 2980, 1670, 1590, 1460, 1330, 1110, 990, 830 cm^{-1}.

HRMS (ESI) m/z: $[M+H]^+$ calcd for $C_{10}H_{11}F_2NO_2{\cdot}H^+$, 216.0831; found: 216.0823.

1-(3,6-dibromopyridin-2-yl)-2-(3,5-difluorophenyl) ethan-1-one (8)

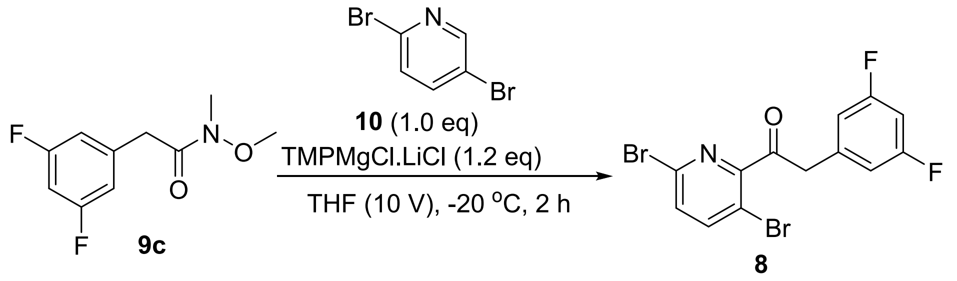

2,5-Dibromopyridine (**10**, 30.0 g, 126.64 mmol, 1 eq) and anhydrous THF (300 mL) were added to a 1 L three-necked round flask equipped with over-head stirrer under N_2 atmosphere. The mixture was cooled to -20 °C and stirred for 10 min. TMPMgCl·LiCl (151.97 mL, 1.0 M, 151.97 mmol, 1.2 eq) was added dropwise. After addition (about 30-40 min), the mixture was stirred at the same temperature for 40 min. A solution of 2-(3,5-difluorophenyl)-N-methoxy-N-methylacetamide **9c** (29.9 g, 139.31 mmol, 1.1 eq) in THF (40 mL) was added dropwise. The resulting mixture was stirred at -20 °C for 1 h and then acidified with 2 M HCl (200 mL) to pH = 1-2. The mixture was then basified to pH = 9-10 with 5 % NaOH (80 mL) and extracted with ethyl acetate (210 mL × 2). The organic layer was combined and washed with brine and evaporated to dryness. The residue was suspended in 5% isopropanol/heptanes (90 mL). The slurry was stirred at rt for 1h. After filtration, the solid was collected and dried to afford **8** (colorless solid, 25 g, 50 % isolated yield, 93% purity by qNMR with 1,3,5-trimethoxybenzene as internal standard). The solid was used for the next step without further purification.

^{1}H NMR (600 MHz, $CDCl_3$): δ 7.83 (d, J = 8.4 Hz, 1H), 7.47 (d, J = 8.4 Hz, 1H), 6.88 – 6.80 (m, 2H), 6.71 (tt, J = 9.0, 2.3 Hz, 1H), 4.39 (s, 2H).

^{13}C{^{1}H} NMR (151 MHz, $CDCl_3$) δ 195.5, 163.0 (dd, J = 248.3, 12.7 Hz), 151.3, 145.0, 139.3, 137.2 (t, J = 9.5 Hz), 131.9, 117.86, 113.0 (dd, J = 20.1, 4.8 Hz), 102.7 (t, J = 25.3 Hz), 45.8.

^{19}F NMR (565 MHz, $CDCl_3$) δ -110.0 (s).

Melting point: 101 °C.

IR (ATR, DCM) v_{max} = 3110, 3070, 2930, 1710, 1590, 1410, 1300, 1110, 990, 820 cm^{-1}.

HRMS m/z: [M+H]$^+$ calcd for $C_{13}H_7Br_2F_2NO·H^+$, 389.8935; found: 389.8923.

1-(3,6-dibromopyridin-2-yl)-2-(3,5-difluorophenyl)ethan-1-ol (7)

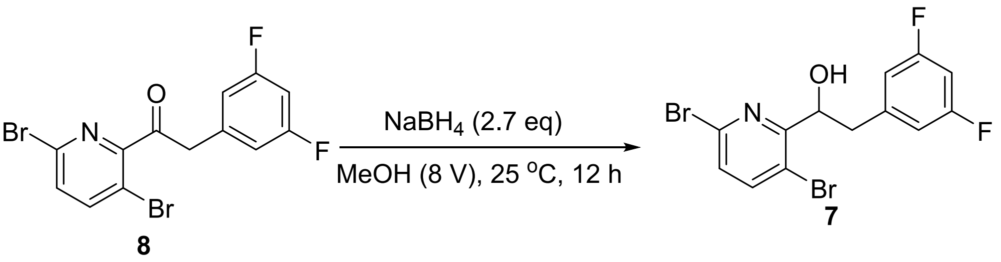

Compound **8** (12 g, 30.79 mmol, 1 eq) and methanol (100 mL) were charged into a 250 mL three-necked round flask equipped with an overhead stirrer. To this mixture sodium borohydride (3.15 g, 81.14 mmol, 2.7 eq) was added in portions over 15 min at 0 °C. The mixture was stirred at 25 °C for 2 h. After completion of the reaction, the solvent was removed under vacuum. The residue was suspended in water (50 mL) and extracted with ethyl acetate (100 mL × 2). The organic layer was combined, washed with brine, dried over Na_2SO_4, and concentrated to afford alcohol **7** as a colorless solid (12 g, 95% isolated yield, 95% qNMR purity with 1,3,5-trimethoxybenzene as internal standard).

^{1}H NMR (600 MHz, $CDCl_3$) δ 7.71 (t, J = 6.9 Hz, 1H), 7.33 (d, J = 8.3 Hz, 1H), 6.76 (dd, J = 8.1, 2.1 Hz, 2H), 6.69 (ddd, J = 9.1, 5.7, 2.3 Hz, 1H), 5.16 (td, J = 8.5, 3.4 Hz, 1H), 3.82 (d, J = 8.9 Hz, 1H), 3.12 (dd, J = 13.9, 3.4 Hz, 1H), 2.82 (dd, J = 13.9, 8.3 Hz, 1H).

^{13}C{^{1}H} NMR (151 MHz, $CDCl_3$): δ 163.0 (dd, J = 248.2, 12.6 Hz), 160.2, 143.1, 141.5, 140.0, 128.8, 118.04, 112.5 (dd, J = 19.7, 5.3 Hz), 102.3 (t, J = 25.4 Hz), 72.2, 43.2.

^{19}F NMR (565 MHz, $CDCl_3$) δ -110.55 (s).
Melting point: 98 °C.

IR (ATR, DCM) v_{max} = 3490, 3090, 3050, 2920, 1590, 1420, 1310, 1110, 1010, 830 cm^{-1}.
HRMS m/z: $[M+H]^+$ calcd for $C_{13}H_9Br_2F_2NO{\cdot}H^+$, 391.9092; found: 391.9064.

1-(3,6-dibromopyridin-2-yl)-2-(3,5-difluorophenyl)ethyl methanesulfonate (11)

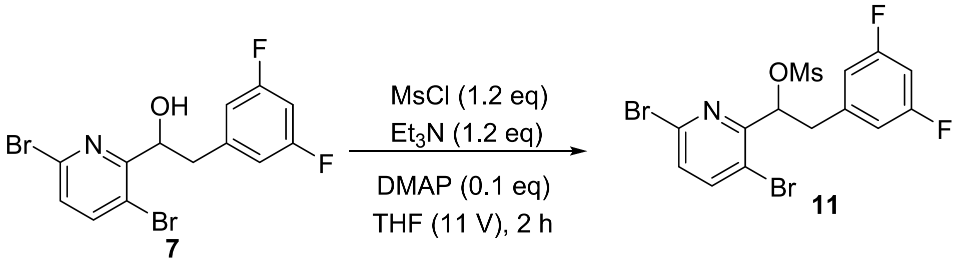

A mixture of alcohol **7** (11.08 g, 28.19 mmol, 1 eq), DMAP (344 mg, 2.82 mmol, 0.1 eq), triethyl amine (3.42 g, 4.72 mL, 33.83 mmol, 1.2 eq), and anhydrous THF (120 mL) was charged into a 250 mL three-neck round bottle equipped with over-head stirrer. To this mixture methanesulfonyl chloride (3.87 g, 2.62 mL, 33.83 mmol, 1.2 eq) was added at 0 °C over 10 min. After addition, the solution was stirred at 25 °C for 2 h. The mixture was quenched with ice-cold water (20 mL) and extracted with ethyl acetate (70 mL × 2). The organic layer was combined, and washed successfully with saturated aq. $NaHCO_3$, brine, dried over Na_2SO_4 and evaporated to dryness to afford a colorless solid **11** (14.16 g, 99% isolated yield, 93% qNMR purity with 1,3,5-trimethoxybenzene as internal standard, containing 2% of EtOAc).

^{1}H NMR (600 MHz, $CDCl_3$): δ 7.70 (d, *J* = 8.3 Hz, 1H), 7.37 (d, *J* = 8.3 Hz, 1H), 6.77 (dd, *J* = 7.8, 2.1 Hz, 2H), 6.73 – 6.67 (m, 1H), 6.14 (dd, *J* = 8.0, 6.1 Hz, 1H), 3.32 (dq, *J* = 13.9, 7.0 Hz, 2H), 2.89 (s, 3H).

^{13}C{^{1}H} NMR (151 MHz, $CDCl_3$): δ 163.0 (dd, *J* = 249.2, 12.8 Hz), 154.8, 143.1, 140.8, 138.9 (t, *J* = 9.3 Hz), 129.9, 119.3, 112.5 (dd, *J* = 19.9, 5.1 Hz), 103.0 (t, *J* = 25.2 Hz), 79.3, 39.9, 39.0.

^{19}F NMR (565 MHz, $CDCl_3$) δ -109.3 (s).
Melting point: 148 °C.

IR (ATR, DCM) v_{max} = 3110, 3060, 3000, 2910, 1595, 1350, 1110, 990, 860, 820 cm^{-1}.
HRMS (ESI) m/z: calcd for $C_{14}H_{12}Br_2F_2NO_3S$ = [M+H$^+$] 469.8867, found 469.8855.

1-(3,6-dibromopyridin-2-yl)-2-(3,5-difluorophenyl)ethan-1-amine (4-*rac*)

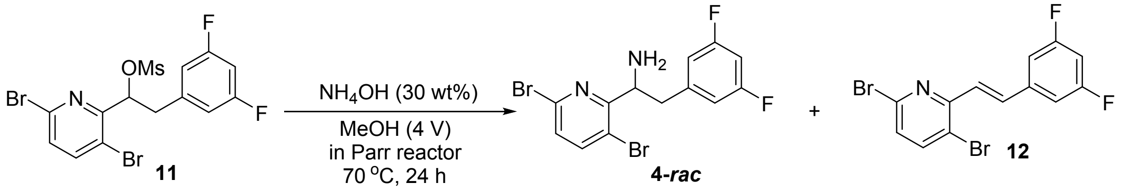

A mixture of compound **11** (10 g, 21.2 mmol, 1 eq), NH_4OH (200 mL, 30%wt), and MeOH (40 mL) were charged into a Parr reactor. The reactor heated at 70 °C for 24 h. After completion of the reaction, the mixture was cooled to 25 °C, and volatiles were removed under reduced pressure. The residue was dissolved in ethyl acetate (100 mL) and the solution was cooled to 0 °C. To this solution aq. HCl (1M, 200 mL) was added at 0 °C. The mixture was stirred for 1h and the organic layer was separated. The aqueous layer was washed with ethyl acetate (50 mL) to remove olefin **12**. The collected aqueous layer was basified with aq. NaOH (10 mL, 50 w/w%) at 0 °C and the

resulting solution was stirred at 25 °C for 1h. The precipitate was collected by filtration and dried to afford off-white solid **4-*rac*** (4.49 g, 54% isolated yield, 99% purity by qNMR with 1,3,5-trimethoxybenzene as internal standard).

Compound **4-*rac***:

^{1}H NMR (600 MHz, $CDCl_3$): δ 7.63 (d, J = 8.2 Hz, 1H), 7.33 – 7.17 (m, 1H), 6.72 (d, J = 6.9 Hz, 2H), 6.67 (t, J = 8.9 Hz, 1H), 4.54 (dd, J = 7.8, 5.5 Hz, 1H), 3.06 (dd, J = 13.5, 5.1 Hz, 1H), 2.76 (dd, J = 13.3, 8.7 Hz, 1H, 1.76 (bs, 2H).

^{13}C{^{1}H} NMR (151 MHz, $CDCl_3$): δ 163.0 (dd, J = 248.0, 13.0 Hz), 162.7, 142.7, 142.5 (t, J = 8.9 Hz), 140.6, 128.2, 119.1, 112.3 (dd, J = 19.7, 4.7 Hz), 102.2 (t, J = 25.4 Hz), 56.1, 43.7.

^{19}F NMR (565 MHz, $CDCl_3$): δ -110.3.

Melting point: 92 °C.

IR (ATR, DCM) v_{max} = 3360, 3030, 3060, 1590, 1490, 1120, 1000, 830 cm^{-1}.

HRMS (ESI) m/z: [M+H^{+}] calcd for $C_{13}H_{10}Br_2F_2N_2 \cdot H^+$, 390.9252; found: 390.9253.

For characterization, compound **12** was purified by column (silica gel, 5%-35% ethyl acetate/heptanes as eluent).

^{1}H NMR (600 MHz, $CDCl_3$): δ 7.68 (d, J = 15.5 Hz, 1H), 7.60 (d, J = 8.3 Hz, 1H), 7.37 (d, J = 15.5 Hz, 1H), 7.14 (d, J = 8.3 Hz, 1H), 7.07 – 6.95 (m, 2H), 6.70 (tt, J = 8.7, 2.2 Hz, 1H).

^{13}C{^{1}H} NMR (151 MHz, $CDCl_3$):163.3 (dd, J = 248.5, 13.0 Hz), 153.4, 142.9, 140.4, 139.4, 135.1, 127.9, 125.6, 120.0, 110.3 (dd, J = 20.2, 5.2 Hz), 104.2 (t, J = 25.6 Hz).

^{19}F NMR (565 MHz, $CDCl_3$): δ -109.7.

Melting point: 112 °C.

IR (ATR, DCM) v_{max} = 3030, 1600, 1590, 1300, 1100, 950, 820 cm^{-1}.

HRMS (ESI) m/z: [M+H^{+}] calcd for $C_{13}H_7Br_2F_2N \cdot H^+$, 373.8986; found: 373.8980.

(*S*)-1-(3,6-dibromopyridin-2-yl)-2-(3,5-difluorophenyl)ethan-1-amine N-acetyl-D-leucine ((*S*)-4-NADL)

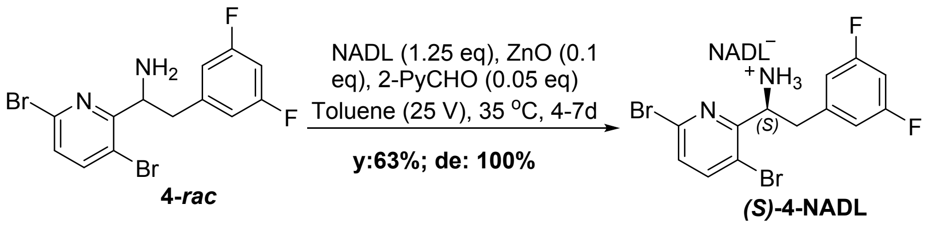

A 500 mL three-necked round bottle flask was equipped with an overhead stirrer, thermocouple, N_2 flow, and water condenser. Amine **4-*rac*** (10 g, 25.5 mmol, 1 eq), N-acetyl-D-leucine (5.5 g, 31.9 mmol, 1.25 eq), ZnO (208 mg, 2.55 mmol, 0.1 eq), anhydrous toluene (250 mL) and 2-pyridine carboxaldehyde (137 mg, 1.28 mmol, 0.05 eq) were charged to the flask under N_2 flow. The reaction mixture was heated to 60 °C and stirred for 6 h, then held at 35 °C. During the course, precipitates were formed after 24h. After completion of the reaction (monitored the wt% of the reaction solution by qNMR, about 4-7d), the mixture was cooled to 25 °C, and the solid was collected by filtration. The filter cake was washed with cold toluene (75 mL × 4). The solid was dried under vacuum at 60 ˚C to afford **(*S*)-4-NADL** as a white solid in 63% yield (11.5 g, 100% de, 79 wt% purity by HPLC).

^{1}H NMR (600 MHz, DMSO-d$_6$): δ 8.02 (d, *J* = 7.6 Hz, 1H), 7.95 (d, *J* = 8.3 Hz, 1H), 7.49 (d, *J* = 8.3 Hz, 1H), 7.02 (t, *J* = 9.4 Hz, 1H), 6.86 (d, *J* = 6.7 Hz, 2H), 4.52 – 4.37 (m, 1H), 4.18 (q, *J* = 7.7 Hz, 1H), 2.94 (dd, *J* = 13.3, 5.9 Hz, 1H), 2.87 (dd, *J* = 13.2, 7.9 Hz, 1H), 1.83 (s, 3H), 1.65 – 1.57 (m, 1H), 1.47 (t, *J* = 7.3 Hz, 2H), 0.88 (d, *J* = 6.6 Hz, 3H), 0.83 (d, *J* = 6.5 Hz, 3H).

^{13}C{^{1}H} NMR (151 MHz, DMSO-d$_6$): δ 174.9, 169.6, 163.3 (d, *J* = 13.4 Hz), 162.9, 161.7 (d, *J* = 13.4 Hz), 143.9, 143.6 (t, *J* = 9.4 Hz), 140.2, 128.7, 119.7, 112.9 (dd, *J* = 19.7, 4.6 Hz), 102.1 (t, *J* = 25.7 Hz), 55.9, 50.9, 42.5, 40.8, 24.8, 23.4, 22.8, 21.9.

^{19}F NMR (565 MHz, DMSO-d$_6$): δ -110.9.

Melting point: 164 °C.

IR (ATR, DCM) ν_{max} = 3360, 3030, 3060, 1590, 1490, 1120, 1000, 830 cm^{-1}.

HPLC (CHIRALPAK IA SFC, CO_2/MeOH = 90/10, flow rate = 2.0 mL/min, detector wavelength = 210 nm) t_R = 4.2 min (0%), 6.8 min (100%)

Specific rotation: $[\alpha]_D^{20}$ = +64.4 (deg·mL·g^{-1}·dm^{-1}) (measured in MeOH (10mg/mL) at 20 ˚C under 589nm)

(*S*)-1-(3,6-dibromopyridin-2-yl)-2-(3,5-difluorophenyl)ethan-1-amine(R)-2-hydroxy-2-phenylacetic acid ((*S*)-4-Mandelate)

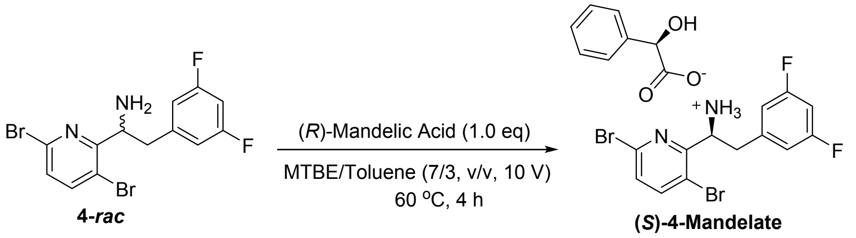

A two-neck 50 mL round bottle flask was charged (*S*)-1-(3,6-dibromopyridin-2-yl)-2-(3,5-difluorophenyl)ethan-1-amine (1.0 g, 2.55 mmol, 1 eq) in MTBE (7 mL) and toluene (3 mL). The mixture was stirred for 10 min at 60 °C. Then (*R*)-2-hydroxy-2-phenylacetic acid ((*R*)-Mandelic acid, 388.10 mg, 2.55 mmol, 1 eq) was added. The reaction mixture was stirred at 60 °C for 4h, then cooled to 0 °C, and aged for 17h. The resulting solid was collected by filtration and the filter cake was washed with a mixed solvent of MTBE/toluene (7/3, v/v, 20V, 20 mL), dried under vacuum at 25 ˚C overnight to afford **(*S*)-4-Mandelate** as a white solid (430 mg, yield: 31%, de: 100%).

^{1}H NMR (600 MHz, DMSO-d$_6$) δ 7.97 (d, *J* = 8.3 Hz, 1H), 7.53 (d, *J* = 8.3 Hz, 1H), 7.39 (d, *J* = 7.4 Hz, 2H), 7.29 (t, *J* = 7.4 Hz, 2H), 7.23 (t, *J* = 7.1 Hz, 1H), 7.05 (t, *J* = 9.2 Hz, 1H), 5.18 (s, 4H), 4.84 (s, 2H), 4.58 (t, *J* = 6.8 Hz, 1H), 3.03 – 2.89 (m, 2H), 2.50 (s, 1H).

^{13}C{^{1}H} NMR (151 MHz, DMSO-d$_6$) δ 174.8, 163.4 (d, *J* = 13.4 Hz), 161.7 (d, *J* = 13.5 Hz), 161.0, 144.1, 141.9, 140.2, 129.2, 128.3, 127.5, 126.9, 119.9, 112.9 (dd, *J* = 19.7, 4.7 Hz), 102.4 (t, *J* = 25.7 Hz), 73.2, 55.32, 41.6.

^{19}F NMR (565 MHz, DMSO-d$_6$): δ -110.7.

Specific rotation: $[\alpha]_D^{20}$ = +31.22 (deg·mL·g^{-1}·dm^{-1}) (measured in MeOH (10mg/mL) at 20 ˚C under 589nm)

Synthesis of (S)-1-(3,6-dibromopyridin-2-yl)-2-(3,5-difluorophenyl)ethan-1-amine ((S)-4)

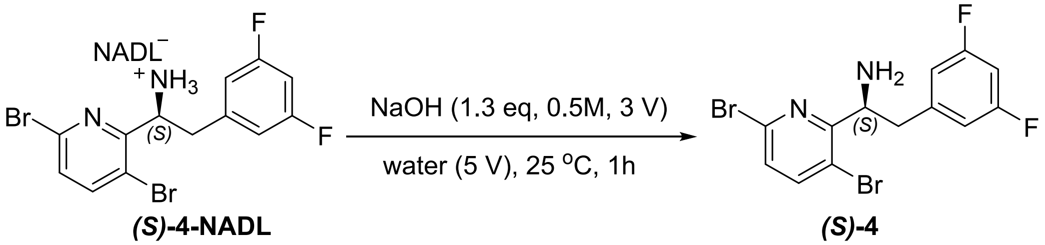

(*S*)-4-NADL (10 g, 12.2 mmol, 1 eq, 80 wt% purity) and water (50 mL, 5V) were charged to a 250 mL three-necked round bottom flask equipped with an overhead stirrer under N_2 atmosphere. To this slurry aq. NaOH (16 mmol, 630 mg, 1.3 eq in 30 mL water, 3V) was added at 20 °C. The reaction mixture was stirred at the same temperature for 1 h. After 1 h, the solid obtained was filtered and dried under vacuum at 25 °C overnight to afford white free amine **(*S*)-4** (5.5 g, 96% isolated yield, 97% wt% by HPLC).

^{1}H NMR (600 MHz, $CDCl_3$): δ 7.65 (d, J = 8.3 Hz, 1H), 7.27 (t, J = 7.9 Hz, 1H), 6.75 (d, J = 6.3 Hz, 2H), 6.68 (dd, J = 12.5, 5.5 Hz, 1H), 4.56 (dd, J = 8.5, 5.3 Hz, 1H), 3.08 (dd, J = 13.5, 5.2 Hz, 1H), 2.78 (dd, J = 13.5, 8.6 Hz, 1H), 1.80 (s, 2H).

^{13}C{^{1}H} NMR (151 MHz, $CDCl_3$): δ 162.9 (dd, J = 248.3, 12.9 Hz), 162.5 (s), 142.6 (s), 142.4 (t, J = 9.1 Hz), 140.5 (s), 128.1 (s), 118.9 (s), 112.2 (dd, J = 19.6, 5.1 Hz), 102.1 (t, J = 25.3 Hz), 56.0, 43.6.

^{19}F NMR (565 MHz, $CDCl_3$): δ -110.2.

Melting Point:114 °C

IR (ATR, DCM) ν_{max} = 3360, 3030, 3060, 1623, 1453, 1123, 998, 857 cm^{-1}.

HPLC (CHIRALPAK IA SFC, CO_2/MeOH = 90/10, flow rate = 2.0 mL/min, detector wavelength = 210 nm) t_R = 4.2 min (0%), 6.8 min (100%)

Specific rotation: $[\alpha]_D^{20}$ = +91.19 (deg·mL·g^{-1}·dm^{-1}) (measured in MeOH (10mg/mL) at 20 °C under 589nm)

NMR Spectra and X-ray data

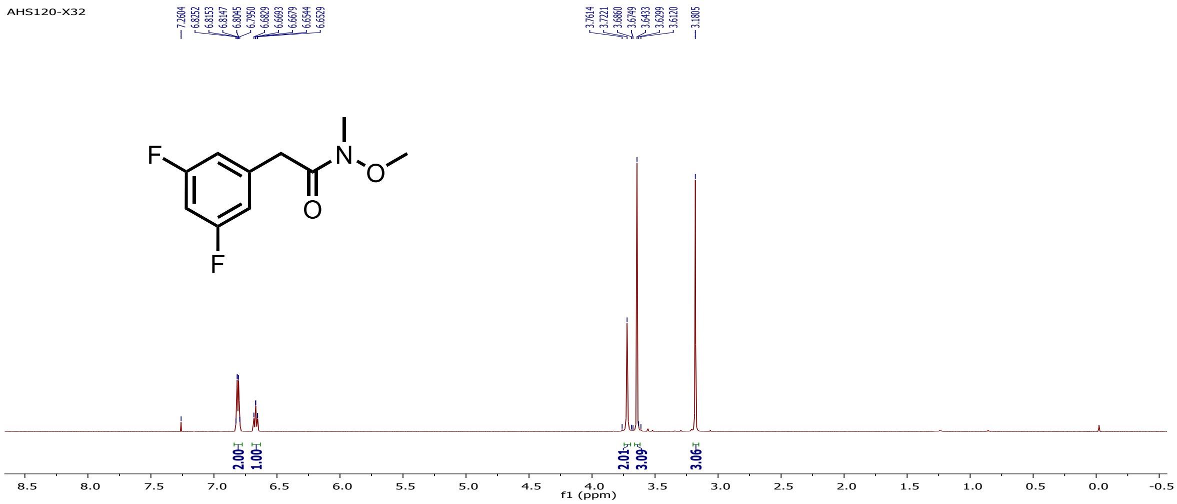

Figure S1. ^{1}HNMR (600 MHz, $CDCl_3$) of Weinreb amide **9c**

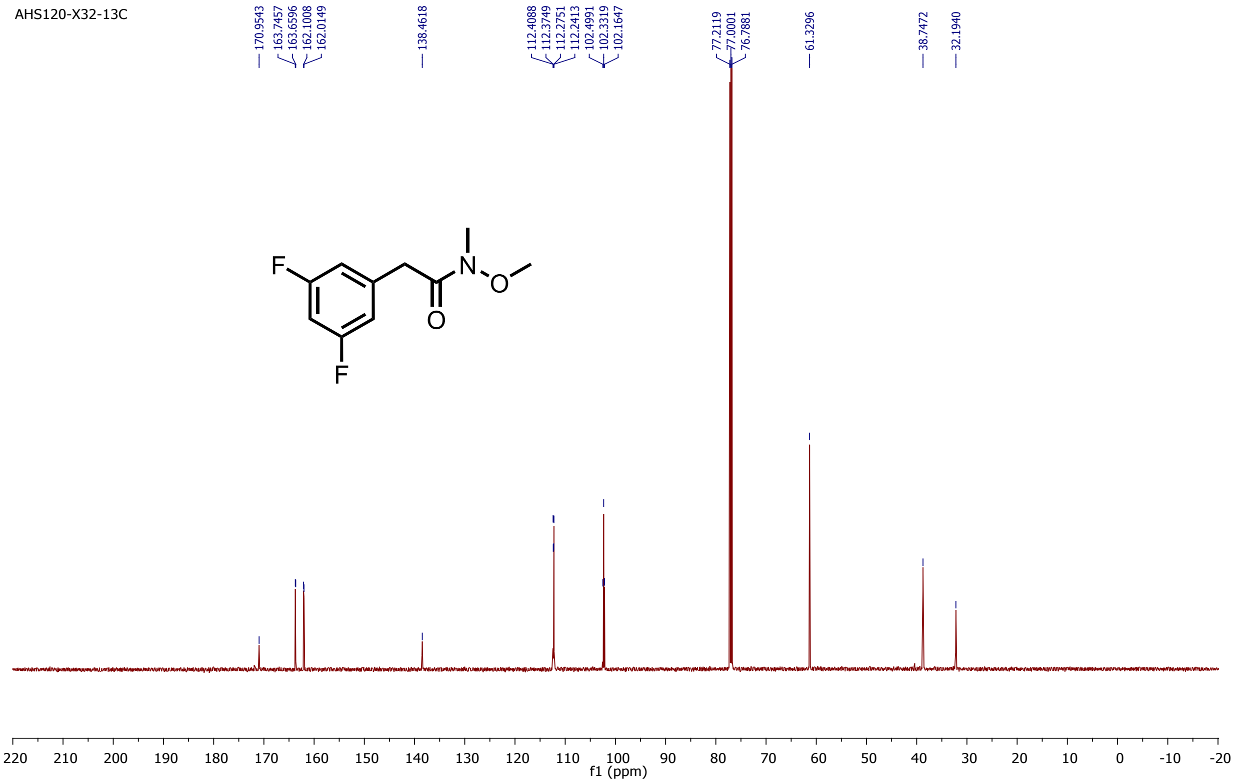

Figure S2. $^{13}C\{^{1}H\}$ NMR (151 MHz, $CDCl_3$) of Weinreb amide **9c**

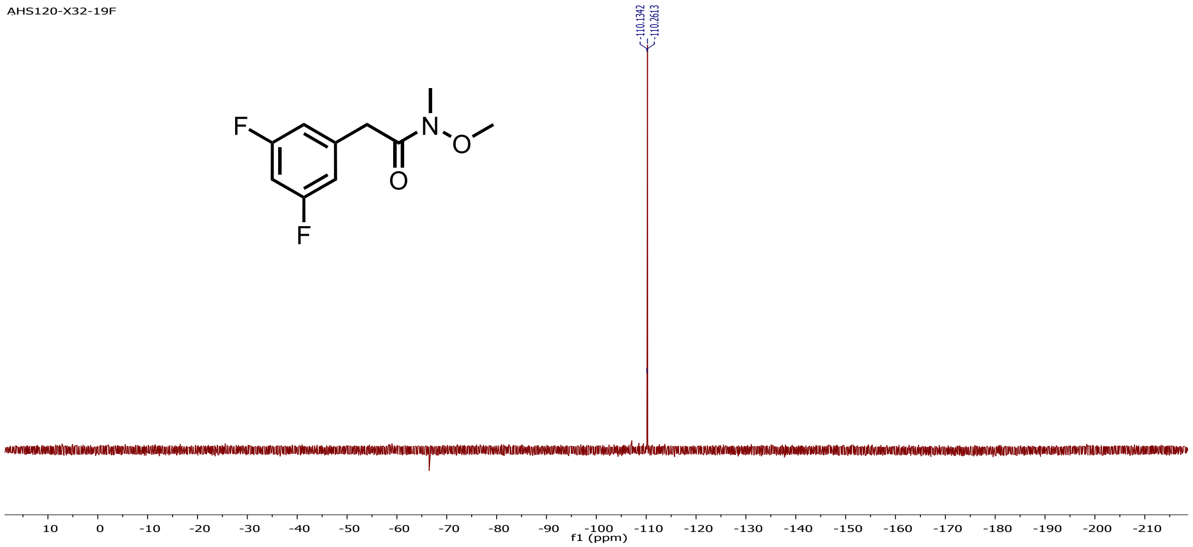

Figure S3. ^{19}F NMR (565 MHz, $CDCl_3$) of Weinreb amide **9c**

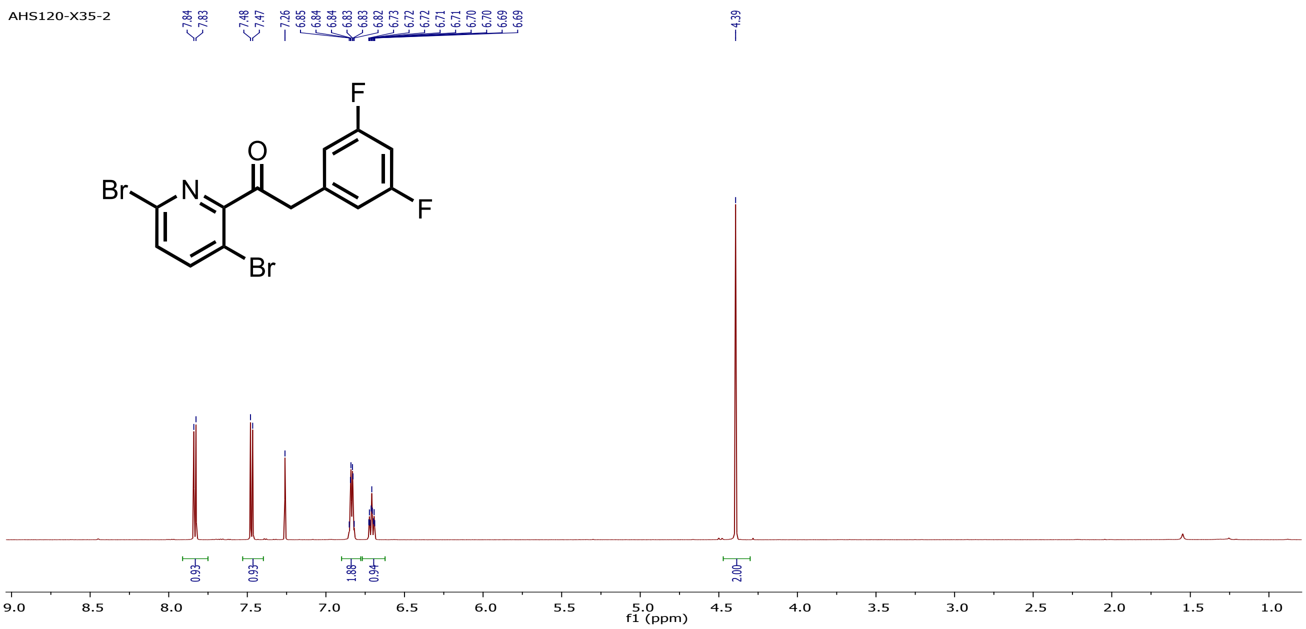

Figure S4. ^{1}HNMR (600 MHz, $CDCl_3$) of ketone **8**

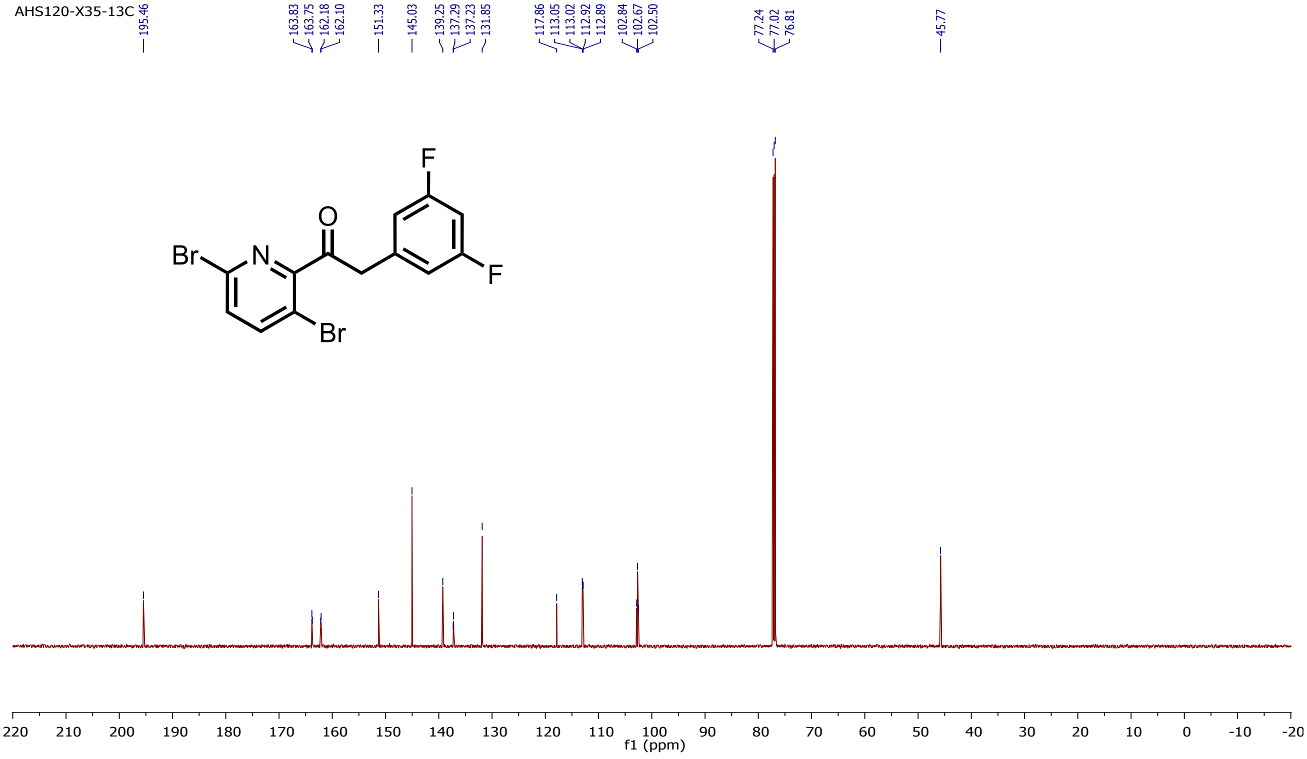

Figure S5. $^{13}C\{^{1}H\}$ NMR (151 MHz, $CDCl_3$) of ketone **8**

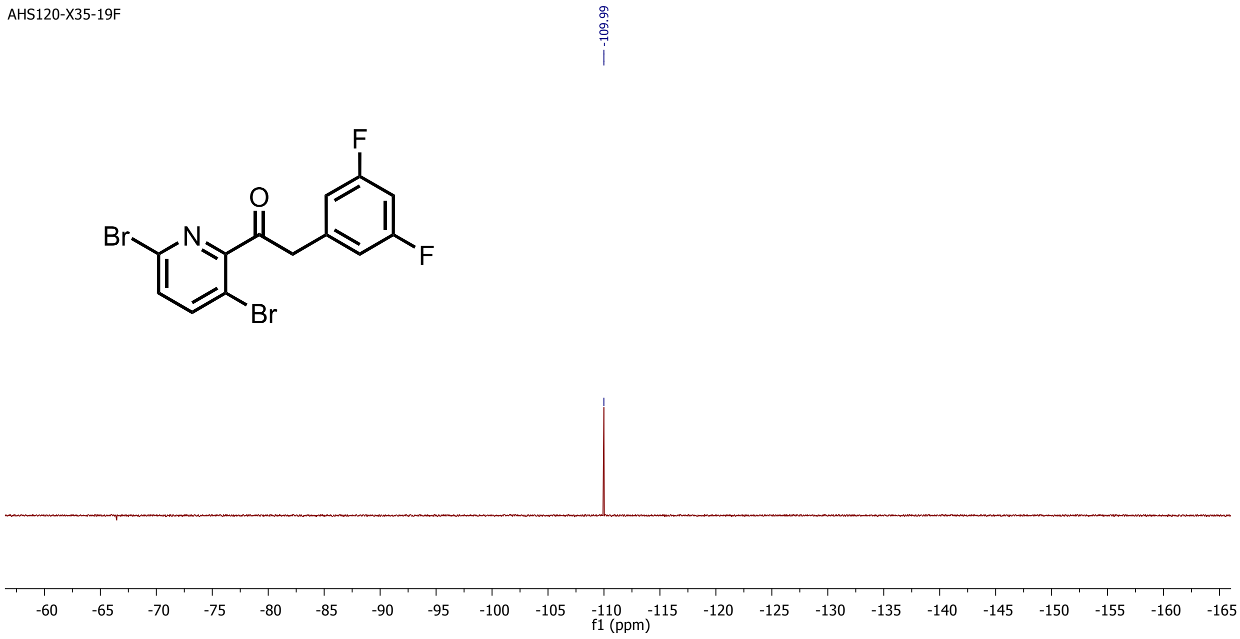

Figure S6. ^{19}F NMR (565 MHz, $CDCl_3$) of ketone **8**

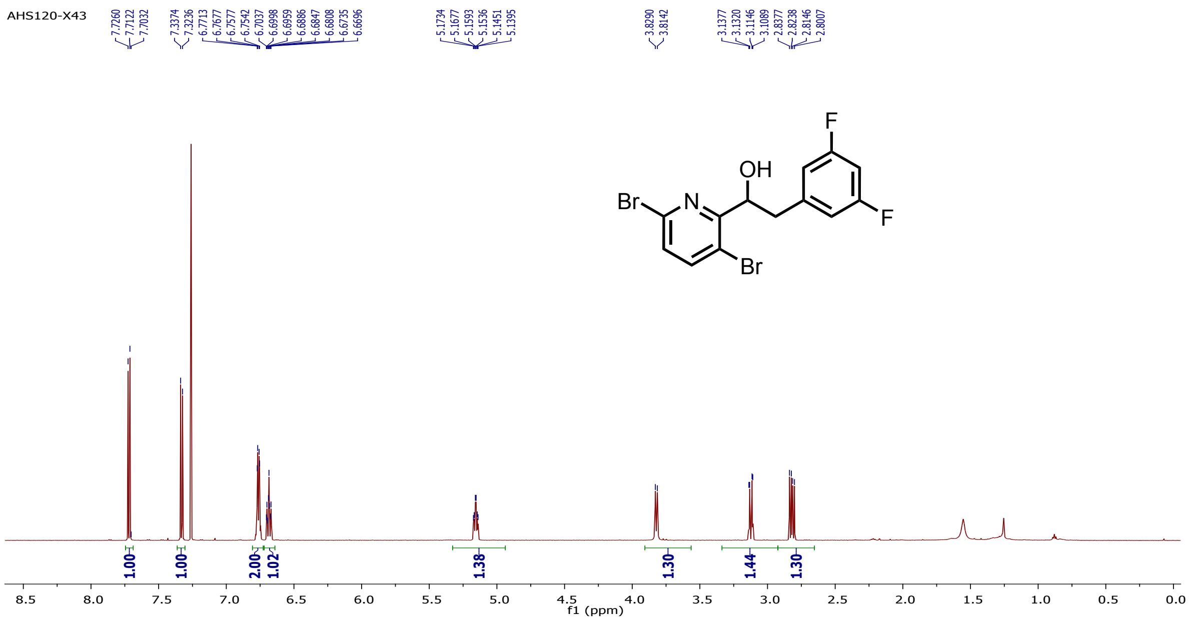

Figure S7. ^{1}HNMR (600 MHz, $CDCl_3$) of alcohol **7**

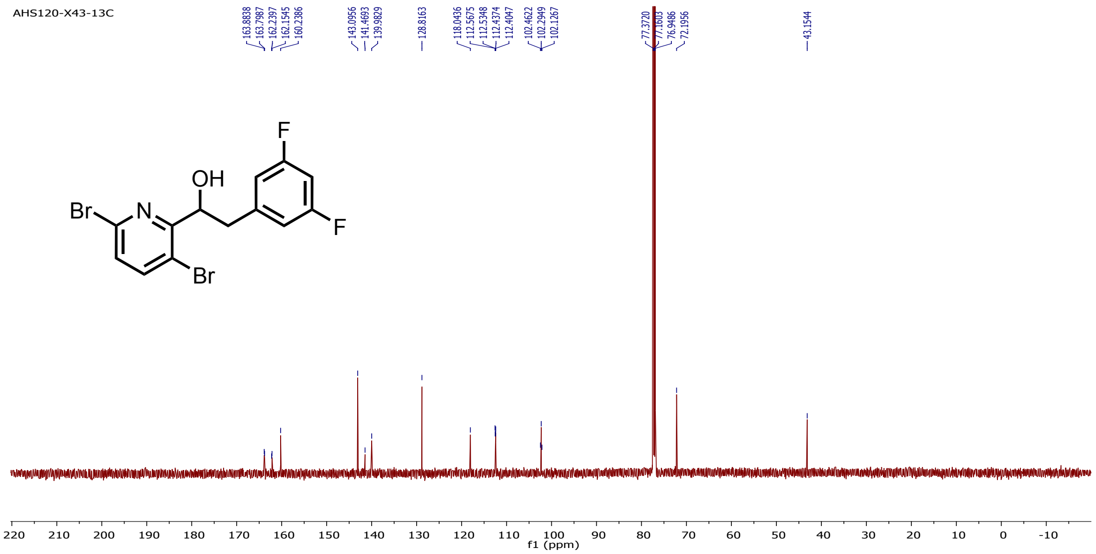

Figure S8. $^{13}C\{^{1}H\}$ NMR (151 MHz, $CDCl_3$) of alcohol **7**

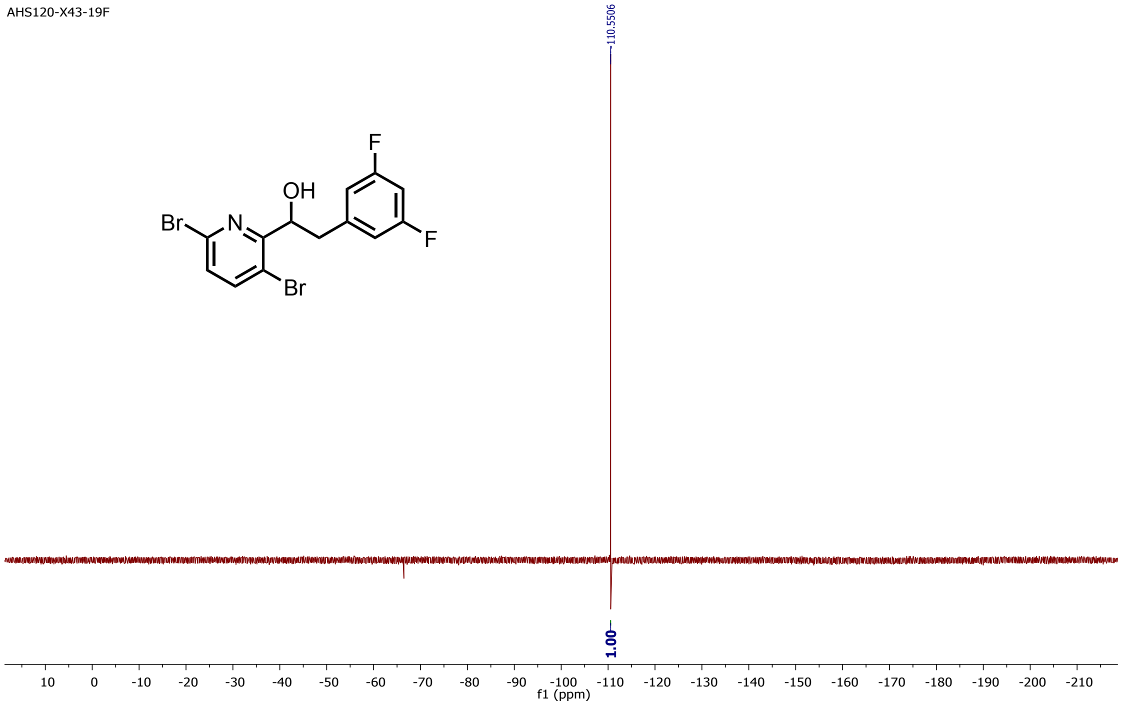

Figure S9. ^{19}F NMR (565 MHz, $CDCl_3$) of alcohol **7**

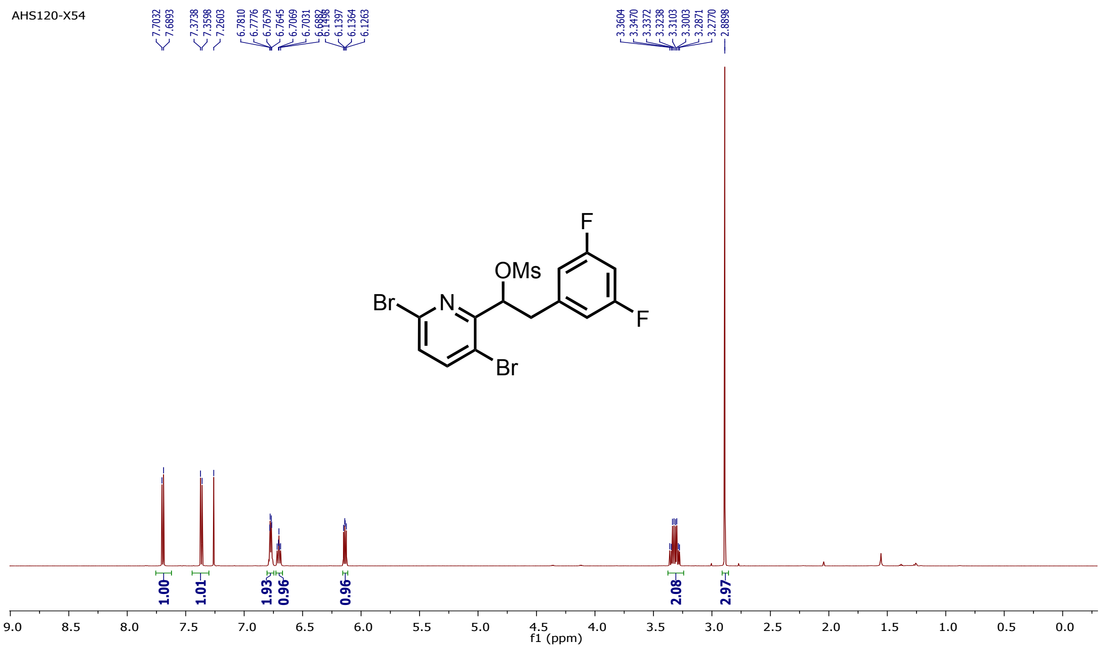

Figure S10. ^{1}HNMR (600 MHz, $CDCl_3$) of compound **11**

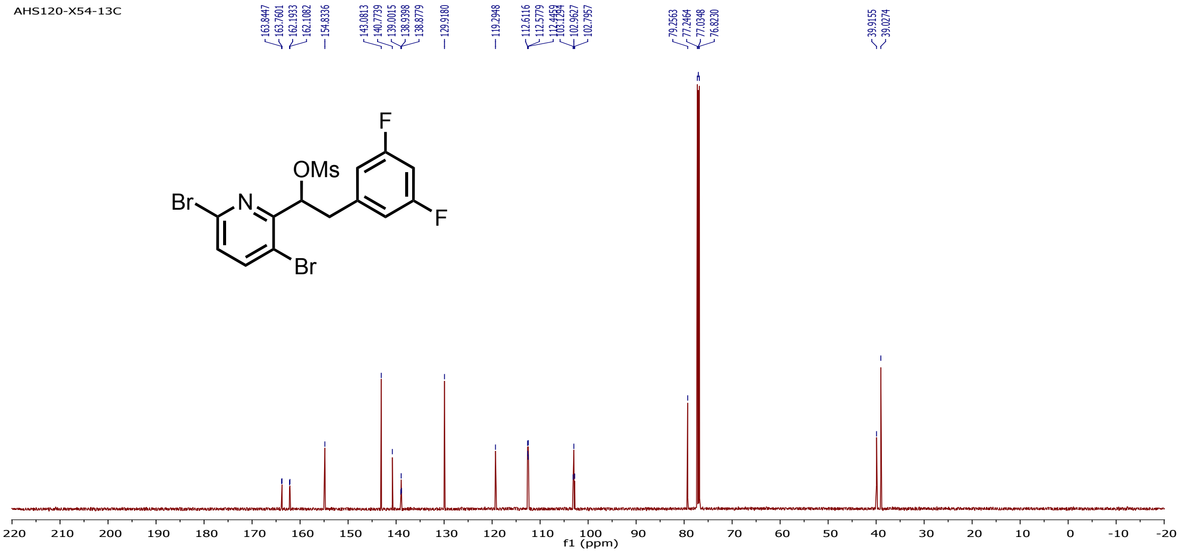

Figure S11. $^{13}C\{^{1}H\}$ NMR (151 MHz, $CDCl_3$) of compound **11**

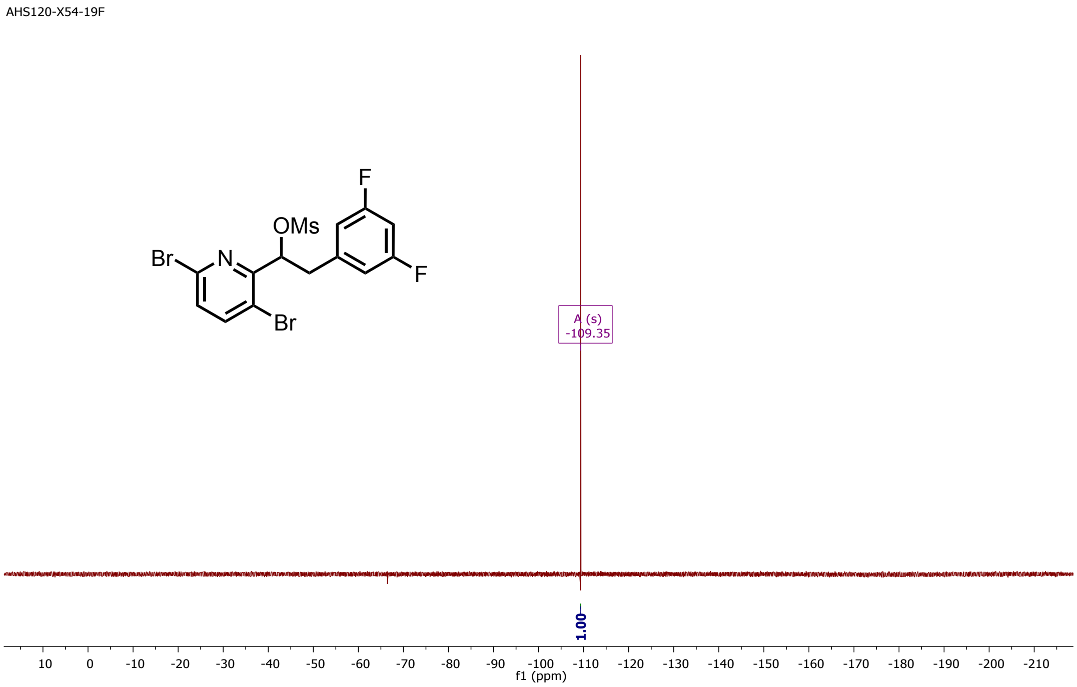

Figure S12. ^{19}FNMR (565 MHz, $CDCl_3$) of compound **11**

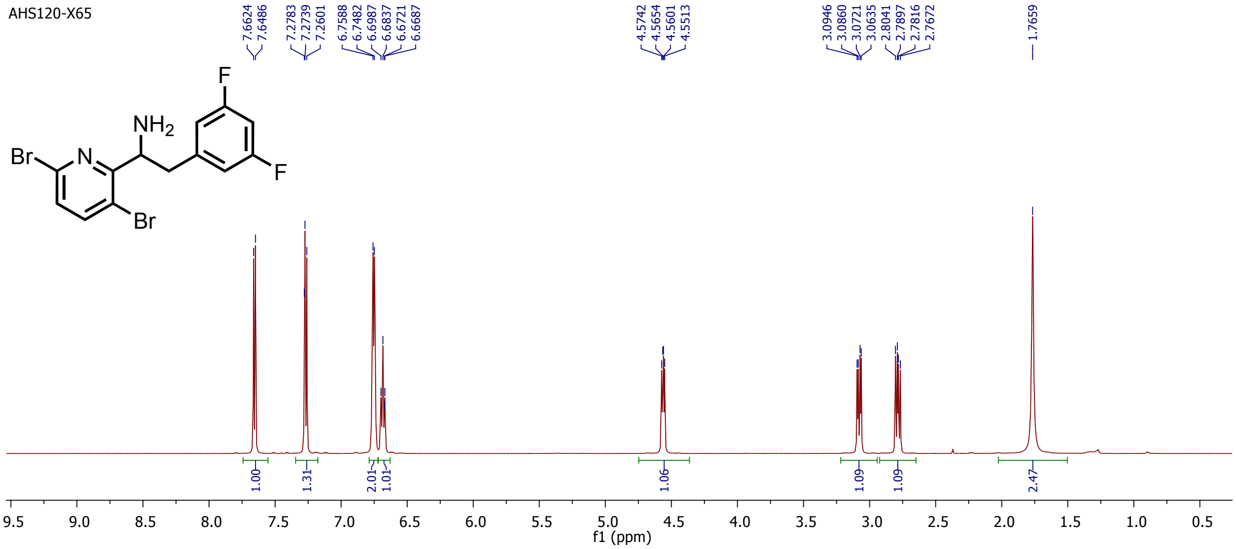

Figure S13. ^{1}HNMR (600 MHz, $CDCl_3$) of amine **4-*rac***

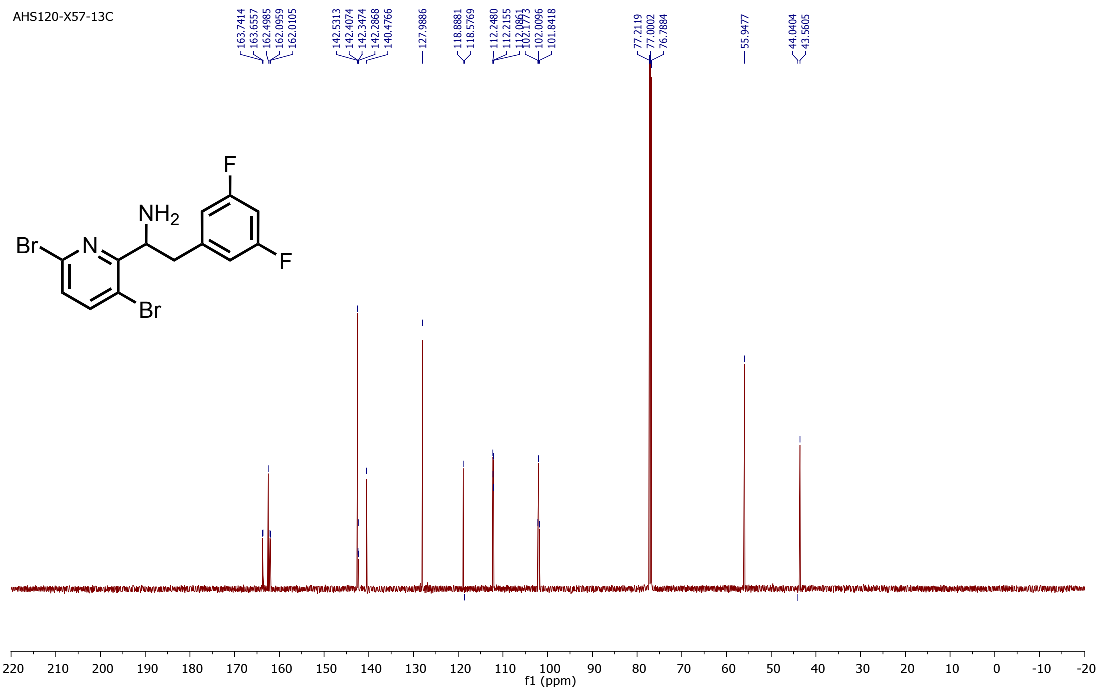

Figure S14. $^{13}C\{^{1}H\}$ NMR (151 MHz, $CDCl_3$) of amine **4-*rac***

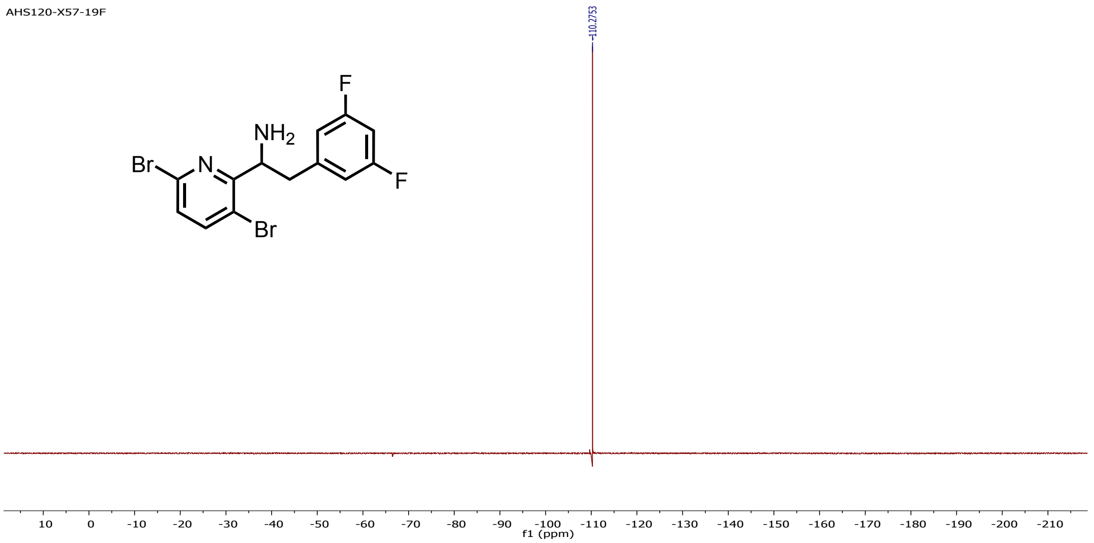

Figure S15. ^{19}FNMR (565 MHz, $CDCl_3$) of amine **4-*rac***

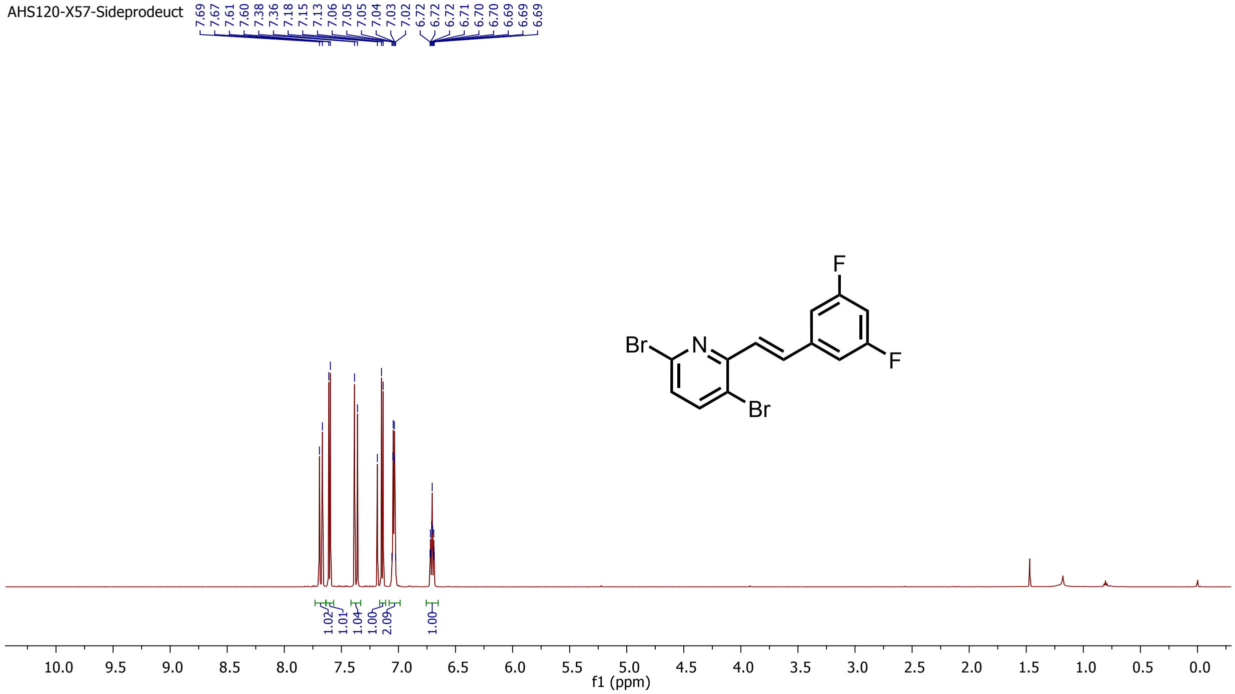

Figure S16. ¹HNMR (600 MHz, $CDCl_3$) of olefin **12**

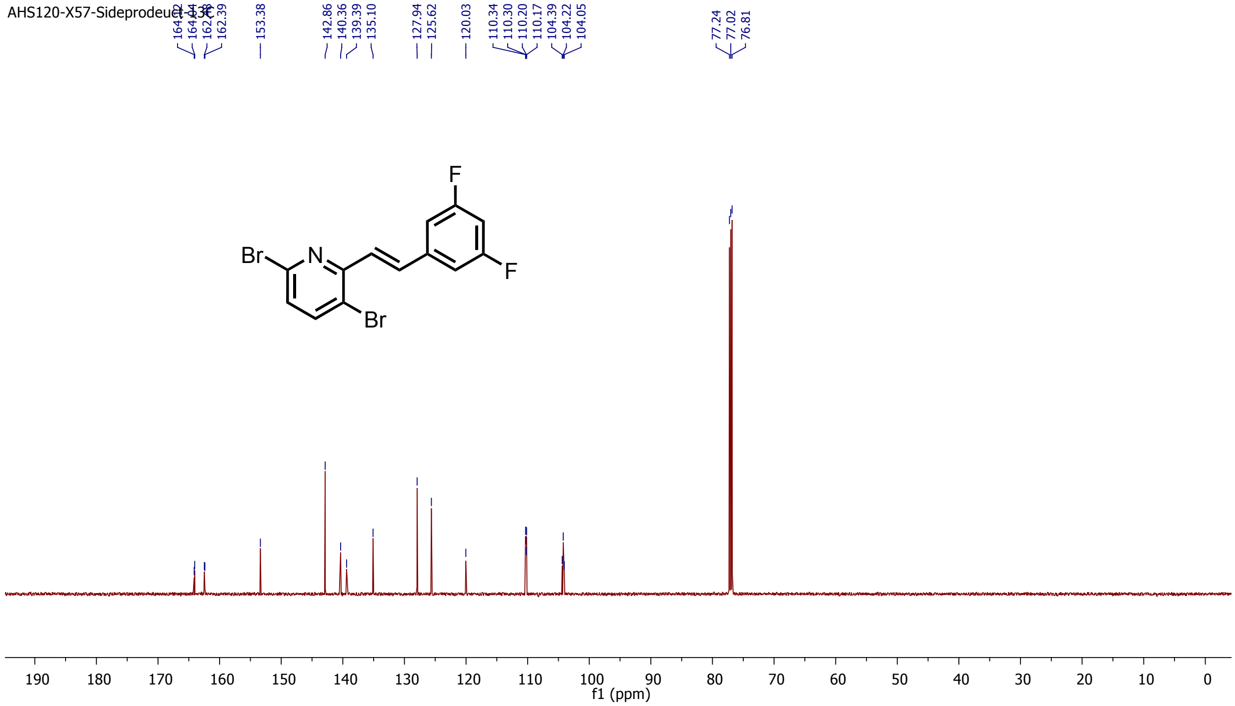

Figure S17. $^{13}C\{^1H\}$ NMR (151 MHz, $CDCl_3$) of olefin **12**

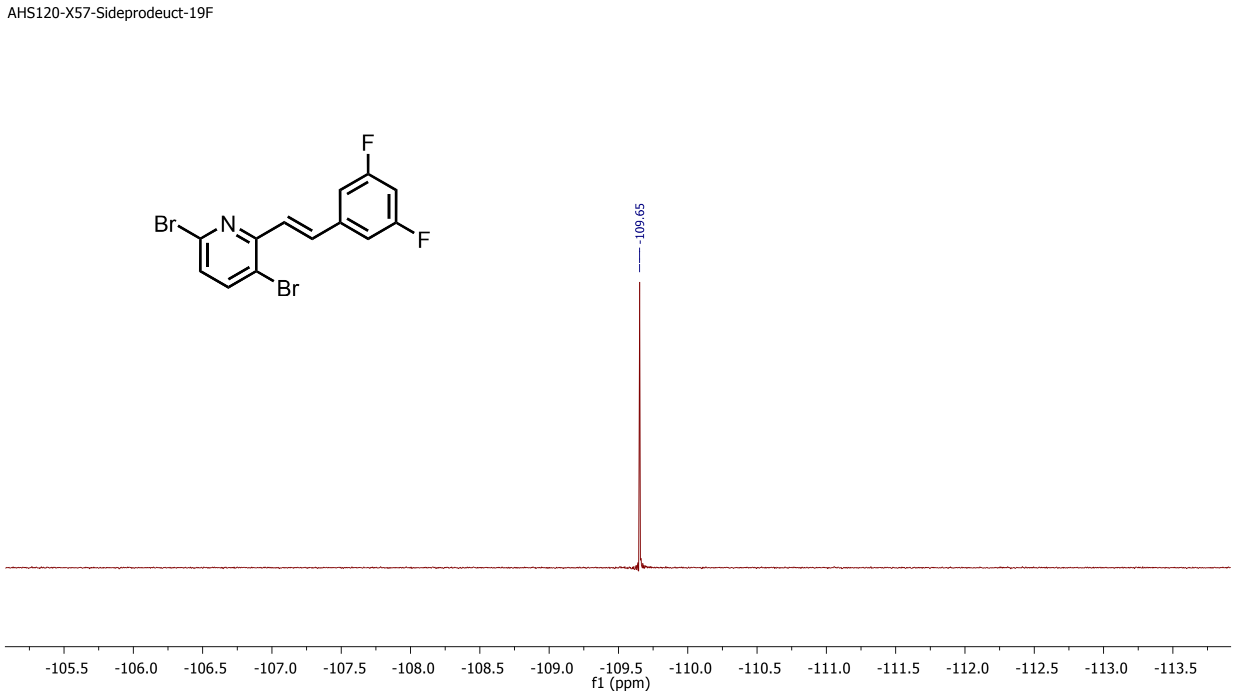

Figure S18. ^{19}FNMR (565 MHz, $CDCl_3$) of olefin **12**

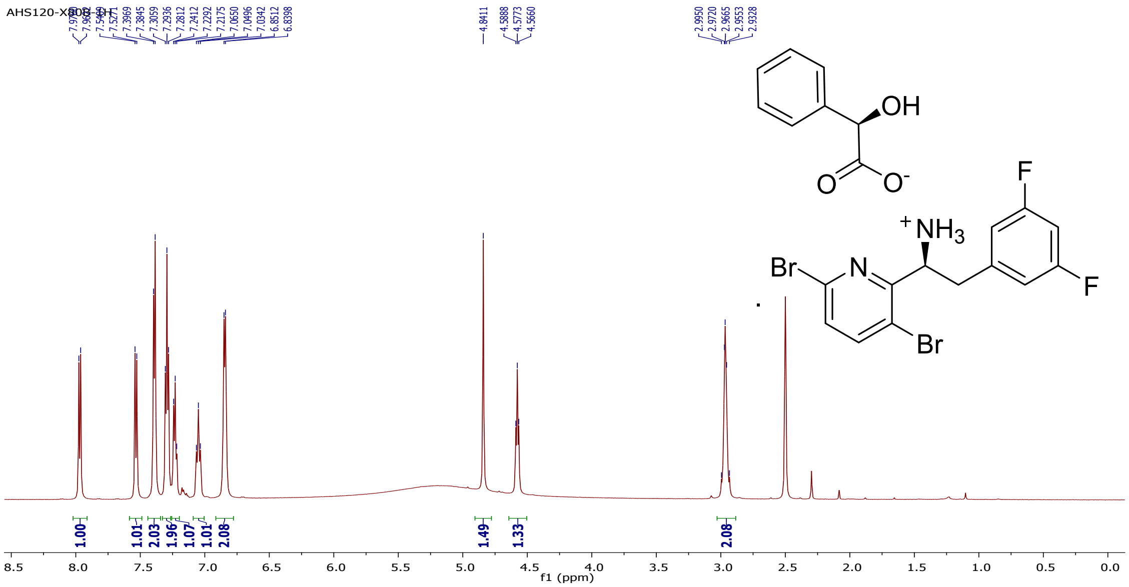

Figure S19. ^{1}HNMR (600 MHz, DMSO-d_6) of **(*S*)-4-Mandelate** Salt

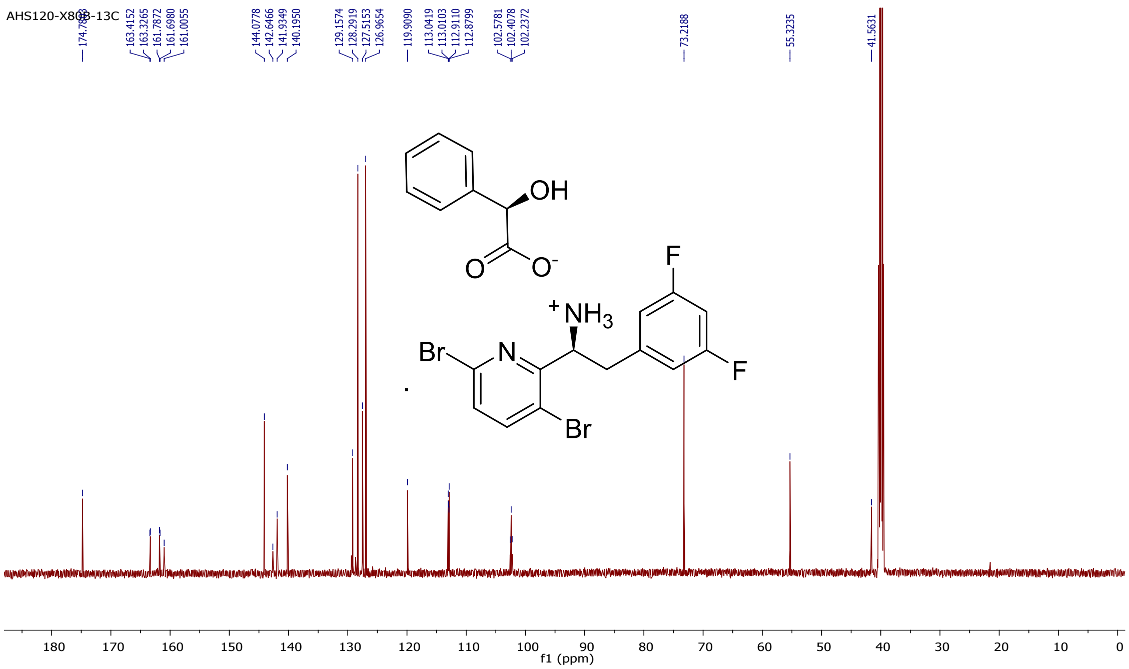

Figure S20. $^{13}C\{^{1}H\}$ NMR (151 MHz, DMSO-d_6) of **(*S*)-4-Mandelate** Salt

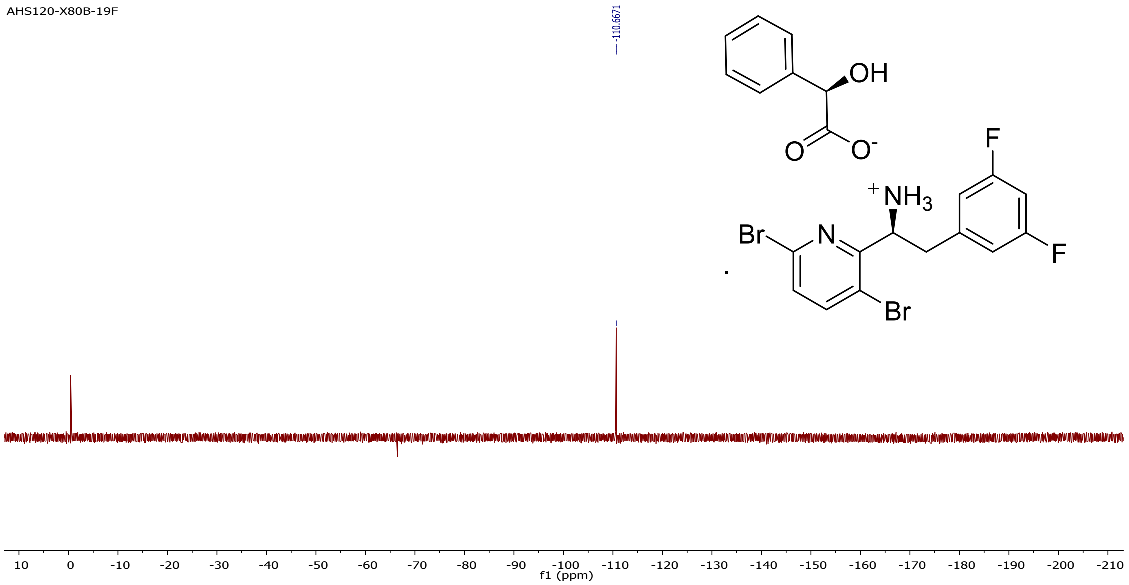

Figure S21. ^{19}FNMR (565 MHz, DMSO-d_6) of **(*S*)-4-Mandelate** Salt

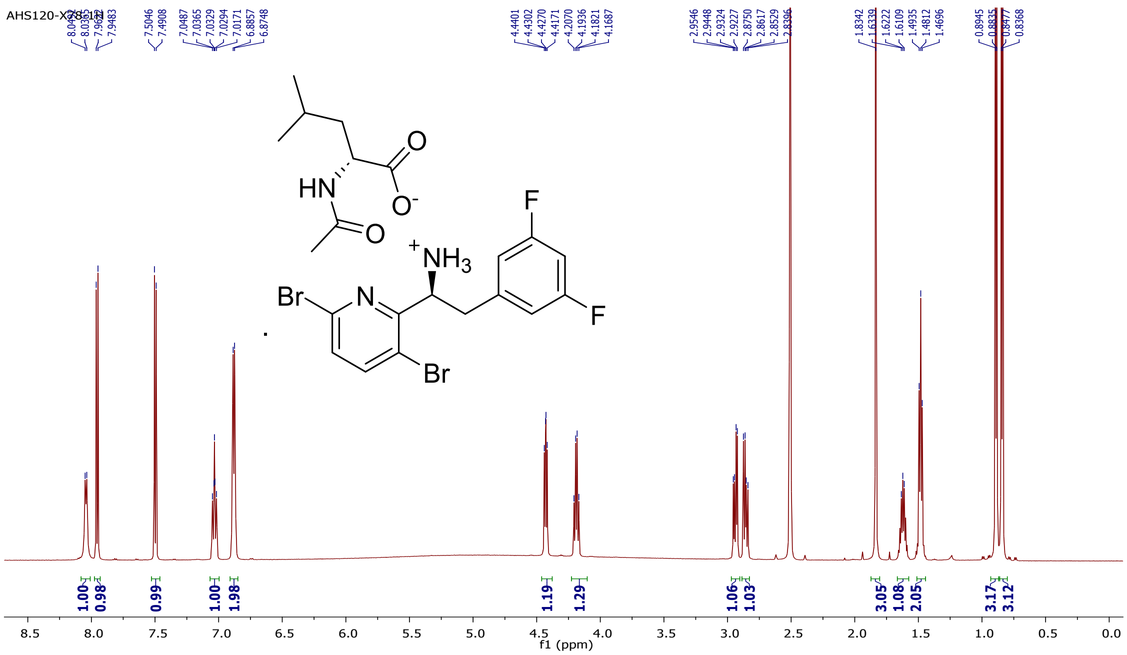

Figure S22. [1] HNMR (600 MHz, DMSO-d_6) of **(*S*)-4-NADL** Leucine Salt

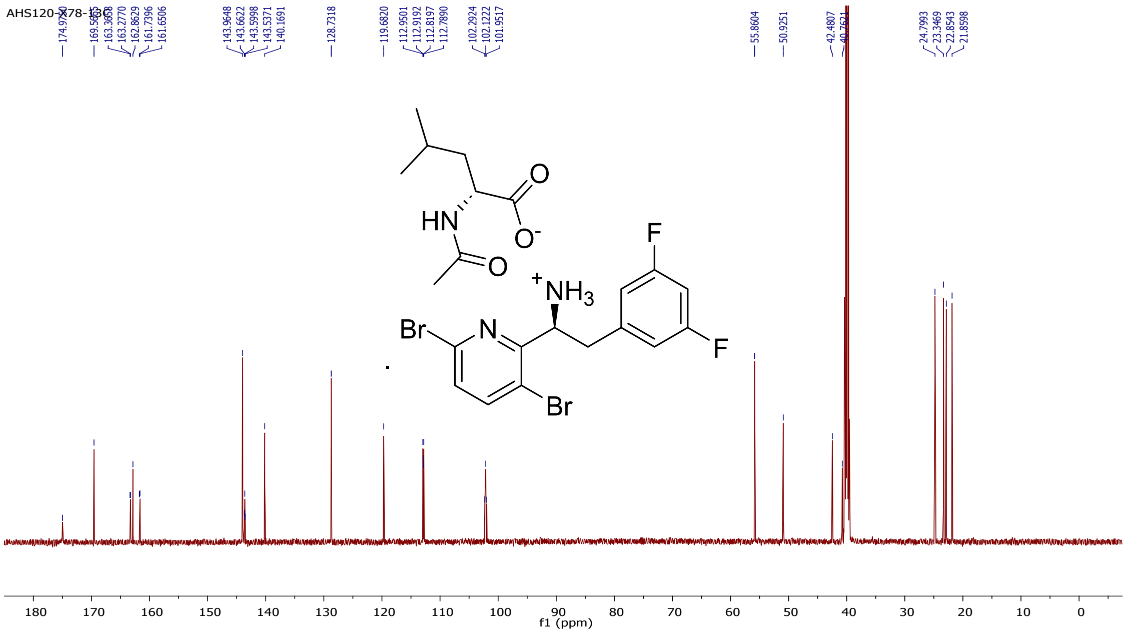

Figure S23. $^{13}C\{^1H\}$ NMR (151 MHz, DMSO-d_6) of **(*S*)-4-NADL** Leucine Salt

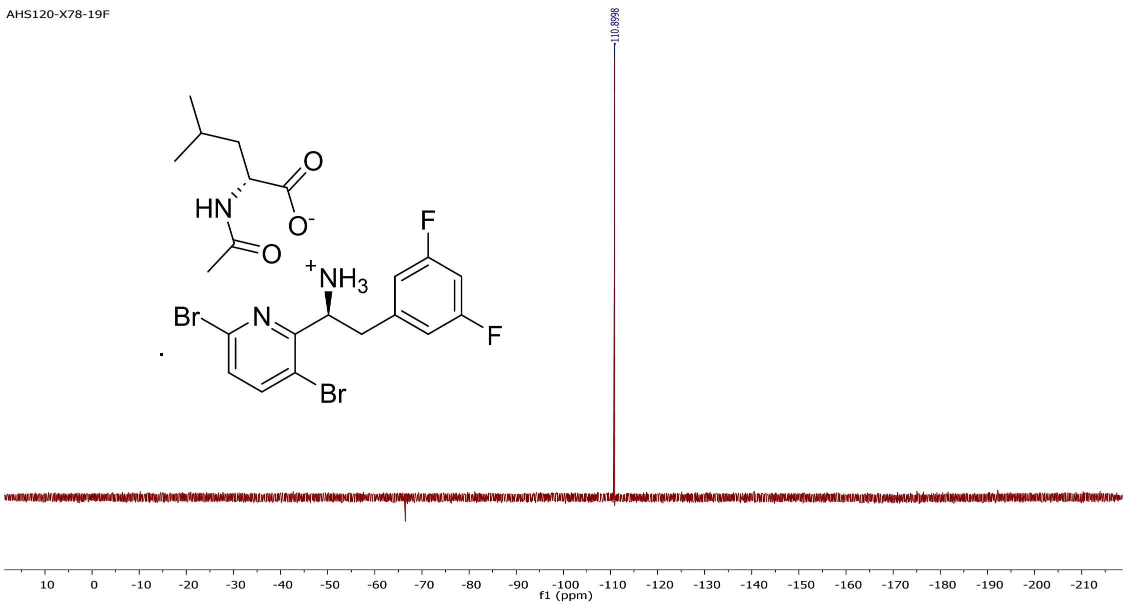

Figure S24. ^{19}F NMR (565 MHz, DMSO-d$_6$) of **(*S*)-4-NADL** Leucine Salt

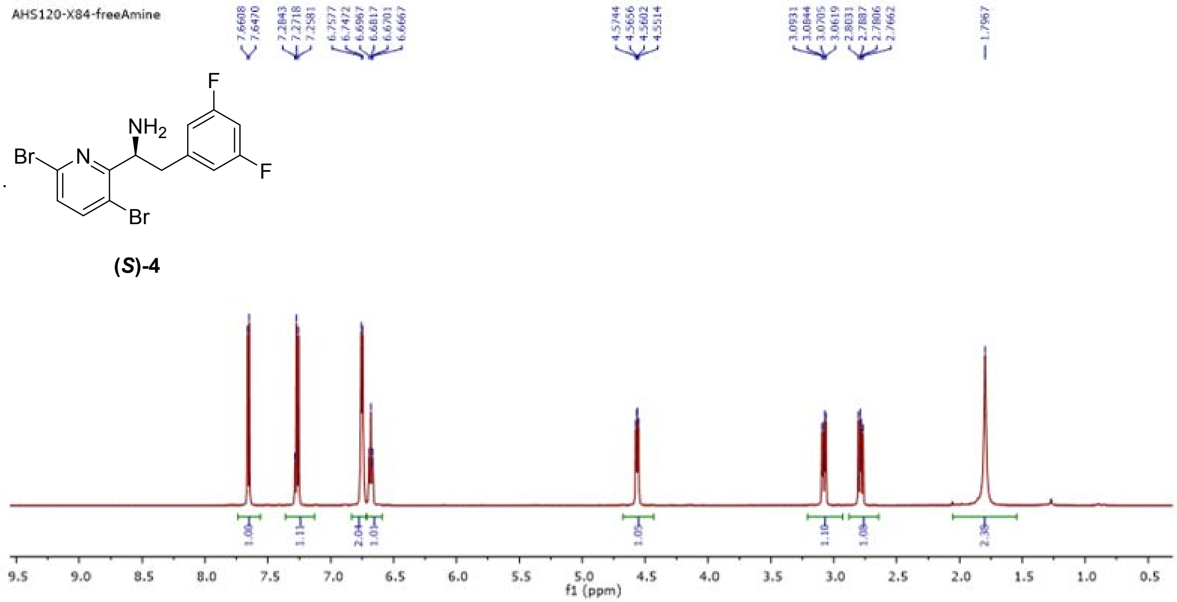

Figure S25. ^{1}HNMR (600 MHz, DMSO-d$_6$) for chiral free amine **(*S*)-4**

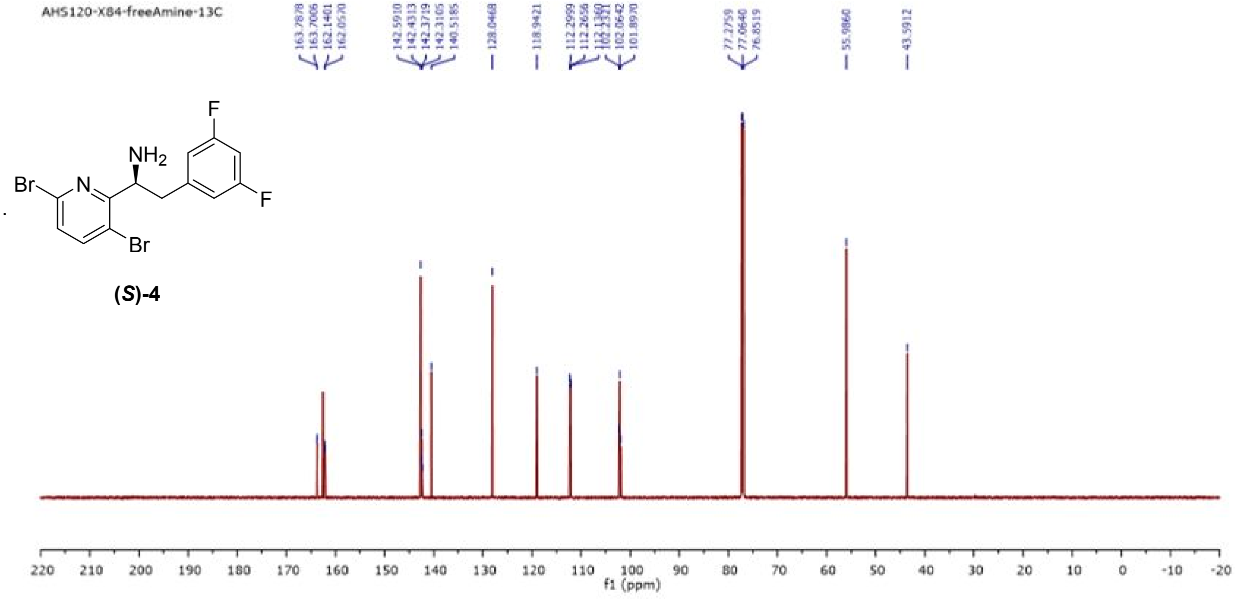

Figure S26. $^{13}C\{^{1}H\}$ NMR (151 MHz, DMSO-d_6) for chiral free amine **(*S*)-4**

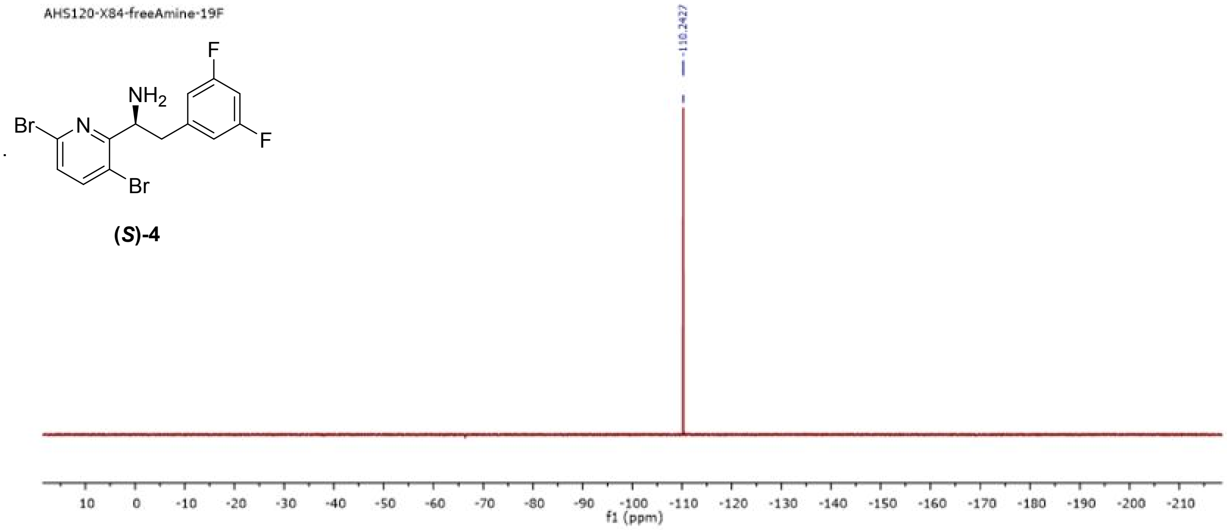

Figure S27. ^{19}FNMR (565 MHz, DMSO-d_6) for chiral free amine **(*S*)-4**

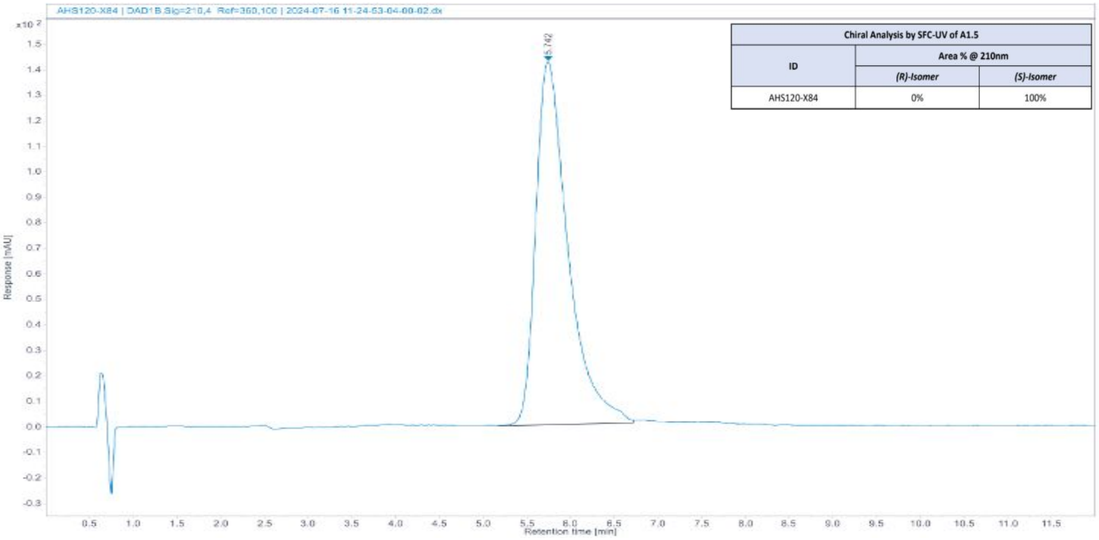

Figure S28. SFC analysis for chiral free amine **(*S*)-4**

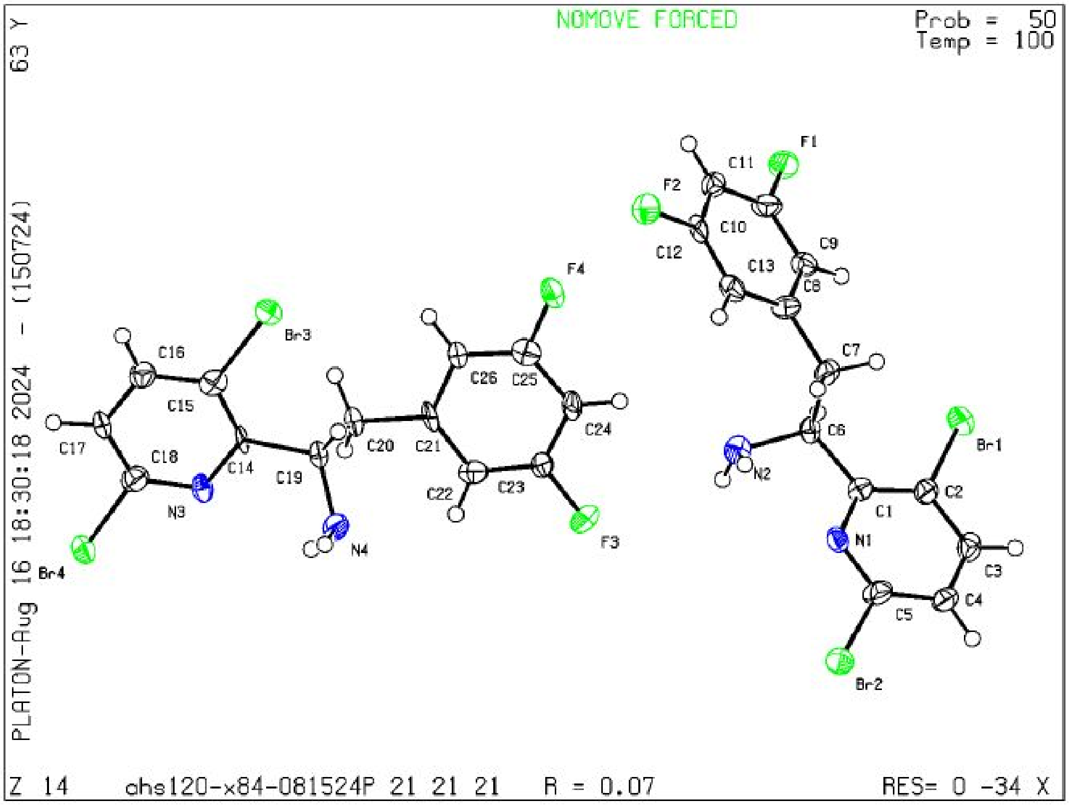

Figure S29. Single X-ray crystal structure data for **(*S*)-4**, thermal ellipsoid drawn at 50% probability

Table S1 Crystal data for **(*S*)-4**

Bond precision:	C-C = 0.0198 A	Wavelength=1.54184	
Cell:	a=4.4017(1)	b=35.5653(6)	c=17.0644(3)
	alpha=90	beta=90	gamma=90
Temperature:	100 K		

	Calculated	Reported
Volume	2671.39(9)	2671.39(9)
Space group	P 21 21 21	P 21 21 21
Hall group	P 2ac 2ab	P 2ac 2ab
Moiety formula	C13 H10 Br2 F2 N2	2(C13 H10 Br2 F2 N2)
Sum formula	C13 H10 Br2 F2 N2	C26 H20 Br4 F4 N4
Mr	392.03	784.10
Dx,g cm-3	1.949	1.950
Z	8	4
Mu (mm-1)	7.821	7.821
F000	1520.0	1520.0
F000’	1512.60	
h,k,lmax	5,43,21	5,43,21
Nref	5292[3130]	5256
Tmin,Tmax	0.363,0.661	0.561,1.000
Tmin’	0.059	

Correction method= # Reported T Limits: Tmin=0.561 Tmax=1.000
AbsCorr = MULTI-SCAN

Data completeness= 1.68/0.99 Theta(max)= 72.098

R(reflections)= 0.0685(5139) wR2(reflections)= 0.1556(5256)

S = 1.092 Npar= 356
